# Elucidating the adsorption mechanism and corrosion inhibition performance of benzothiazolium salts on carbon steel in acidic solution

**DOI:** 10.1039/d5ra07709e

**Published:** 2025-11-24

**Authors:** A. Barrahi, M. E. M. Mekhzoum, M. Benkaddour, M. El Faydy, B. Dikici, R. Bouhfid, Ahmed A. Farag, I. Warad, F. Bentiss, A. Zarrouk

**Affiliations:** a Laboratory of Molecular Spectroscopy Modelling, Materials, Nanomaterials, Water and Environment, CERNE2D, Faculty of Sciences, Mohammed V University in Rabat Morocco asmaabarrahi@gmail.com azarrouk@gmail.com +212697474666; b Moroccan Foundation for Advanced Science, Innovation and Research (MAScIR), Composites and Nanocomposites Center, Rabat Design Center Rue Mohamed El Jazouli, Madinat El Irfane 10100 Rabat Morocco; c Mohammed V University in Rabat Rabat Morocco; d Laboratory of Applied Chemistry and Environment (LCAE), Faculty of Sciences, Mohammed First University 60000 Oujda Morocco; e Department of Mechanical Engineering, Ataturk University 25240 Erzurum Türkiye; f Mohammed VI Polytechnic University Lot 660 Hay Moulay Rachid Ben Guerir 43150 Morocco; g Egyptian Petroleum Research Institute (EPRI) Cairo 11727 Egypt; h Department of Chemistry, AN-Najah National University P.O. Box 7 Nablus Palestine; i Laboratoy of Catalysis and Corrosion of Materials, Faculty of Sciences, Chouaib Doukkali University PO Box 20 M-24000 El Jadida Morocco

## Abstract

This study evaluates the corrosion inhibition performance of two benzothiazolium salts, (*E*)-2-(4-bromostyryl)-3-ethylbenzo[*d*]thiazol-3-ium iodide (BBEI) and (*E*)-2-(2-chlorostyryl)-3-ethylbenzo[*d*]thiazol-3-ium iodide (BCEI), on carbon steel (CS) in 1 M HCl solution. The inhibitory action was investigated using potentiodynamic polarization (PDP) and electrochemical impedance spectroscopy (EIS). Results indicated that the inhibition efficiency of both compounds increased with concentration but decreased with temperature, reaching maximum values of 98.6% for BBEI and 96.9% for BCEI at 10^−3^ M and 303 K. PDP analyses revealed that both molecules act as mixed-type inhibitors, suppressing anodic dissolution and cathodic hydrogen evolution. Adsorption studies showed good agreement with the Langmuir isotherm, suggesting the formation of stable monolayer coverage on the CS surface. Surface characterization techniques, including UV-Vis, SEM/EDS, AFM, XRD, contact angle measurement, XPS and FTIR, confirmed the development of a protective inhibitor film that significantly reduced metal dissolution. The observed protective layers correlated with the high inhibition efficiencies recorded electrochemically. To complement the experimental findings, theoretical investigations were performed using density functional theory (DFT) and molecular dynamics (MD) simulations. Theoretical descriptors, adsorption energies, and electronic parameters highlighted the strong affinity of BBEI and BCEI towards the steel surface, corroborating the experimental results. Overall, the combined experimental and theoretical approach provides a comprehensive understanding of the inhibition mechanism of benzothiazolium salts on carbon steel in acidic medium, demonstrating their potential as effective corrosion inhibitors for practical applications.

## Abstract 2

BBEI and BCEI provide up to 98.6% corrosion protection for carbon steel in 1 M HCl. Electrochemical, surface, DFT and MD results confirm strong adsorption and efficient inhibition.

## Graphical abstract

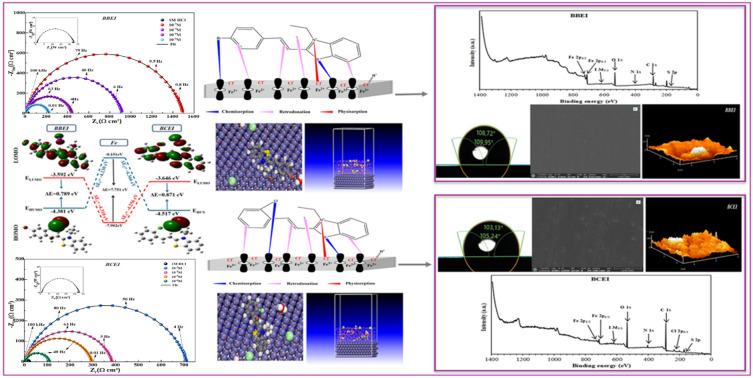

## 1. Introduction

Metal corrosion has long been acknowledged as a significant economic phenomenon. The process of corrosion is the deterioration of a metal or material surface resulting from chemicals reacting with the medium.^1^ It might cause the metal surface to erode and get worn out. Corrosion can be prevented *via* several methods, including by designing appropriate metal surfaces, utilizing protective coatings, using inhibitors, and using cathodic protection. Steel, the most commonly used metal, is significantly impacted by corrosion, which unquestionably severely affects the lifespan of metals.^2,3^ It is well known that carbon steels are aggressively degraded when exposed to corrosive conditions like acidic solutions, which leads to oxide formation on their surface. Carbon steel is a material preferred for a variety of applications in numerous sectors due to its exceptional chemical characteristics, quality, and versatility.^4,5^ The damaging impacts of corrosion on alloys and metals have been combated through several initiatives. While there are several ways to control this phenomenon, one of the more successful approaches is the use of corrosion inhibitors.^6,7^ According to a survey of the available literature on inhibitors of corrosion, the majority of organic inhibitors contain substances like nitrogen, oxygen, sulfur, and/or delocalized p-electrons, which allow the molecules to cling to metal surfaces and halt corrosion.^8,9^

Benzothiazoles are a well-known class of heterocyclic compounds that include two heteroatoms (S and N). They display a variety of pharmaceutical properties, including antibacterial, anticancer, anti-inflammatory, analgesic, and antidiabetic.^10,11^ Additionally, we discovered a previously unreported organic corrosion inhibitor that we may utilize to effectively prevent corrosion of CS substrate.^12,13^ This inhibitor creates a very strong anti-corrosion barrier.

By comparing the protective properties of two types of benzothiazolium salts, namely (*E*)-2-(4-bromostyryl)-3-ethylbenzo[*d*]thiazol-3-ium iodide (BBEI) and (*E*)-2-(2-chlorostyryl)-3-ethylbenzo[*d*]thiazol-3-ium iodide (BCEI). These analogs function as steel corrosion inhibitors in HCl electrolytes. BBEI and BCEI inhibitory effectiveness was evaluated using potentiodynamic polarization measurements (PDP) and electrochemical impedance spectroscopy (EIS) techniques and surface analyses. Quantum chemical calculations were also used to clarify the connection between the molecular structures and the inhibitory effectiveness of benzothiazole compounds.

## 2. Materials and procedures

### 2.1. Tested compounds

**Fig. 1:**
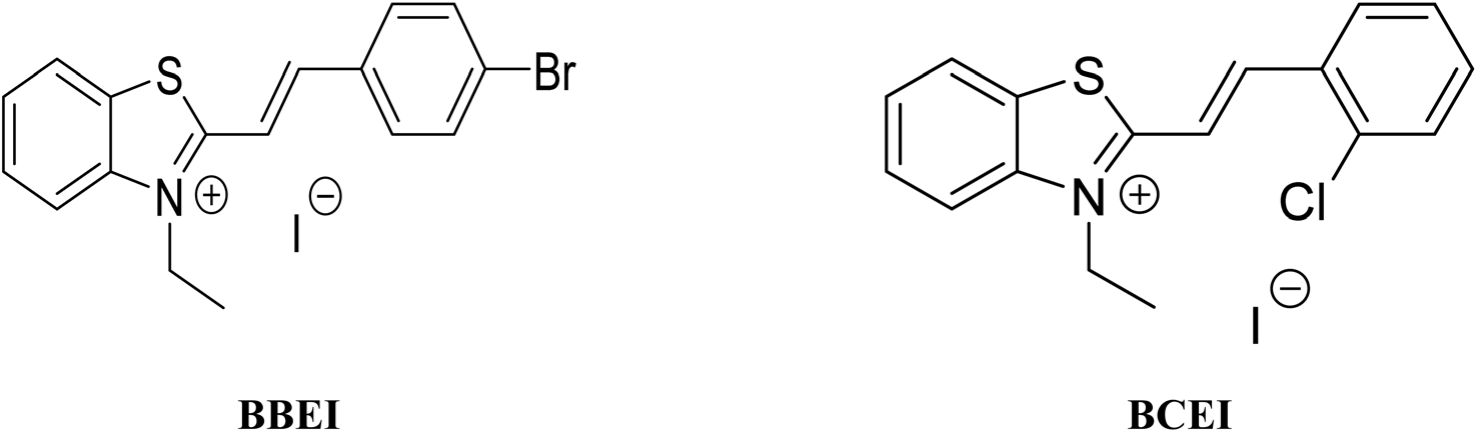
The molecule structures of BBEI and BCEI.

The chemical compound benzothiazole has a heterocyclic ring of a benzene nucleus joined to a thiazole nucleus. In many different contexts, including medicinal chemistry for drug development and environmental chemistry for corrosion management, certain benzothiazole compounds have been investigated for their ability as inhibitors. The two exam-inhibitors, namely (*E*)-2-(4-bromostyryl)-3 ethylbenzo[*d*]thiazol-3-ium iodide (BBEI) and (*E*)-2-(2-chlorostyryl)-3-ethylbenzo[*d*]thiazol-3-ium iodide (BCEI) whose chemical structures are shown in Fig. 1. The two test inhibitors, (*E*)-2-(4-bromostyryl)-3-ethylbenzo[*d*]thiazol-3-ium iodide (BBEI) and (*E*)-2-(2-chlorostyryl)-3-ethylbenzo[*d*]thiazol-3-ium iodide (BCEI) whose chemical structures are provided in Fig. 1.

### 2.2. Solution

Electrochemical measurements were made using a test solution of 1 M hydrochloric acid (HCl) solution, which was created by diluting the hydrochloric acid concentrate with distilled water to evaluate the effectiveness of the inhibitor in preventing corrosion. The necessary quantities of the inhibitor, varying between 10^−3^ and 10^−6^ M, were added directly to the 1 M HCl solution and agitated until completely dissolved. A stock solution of 1 M HCl contains 0.001 M of inhibitor was created by dissolving the necessary quantity of inhibitor in the HCl solution. The initial solution was diluted with the required volume of HCl solution to create different concentrations. The experiments were performed at 303 K.

### 2.3. Material

Before starting each experiment, the working electrode had a pre-treatment process that involved cleaning its surface with abrasive paper of varied degrees of granularity (SiC 180, 320, 400, 600 and 1200). This was done to ensure reliable and consistent results. After that, distilled water was used to rinse the electrode before it was dried. The materials utilized for this research as a working electrode was CS with the following composition (wt%) of C (0.3700%); Si (0.2300%); Mn (0.6800%); S (0.0160%); Cr (0.0770%); Ti (0.0110%); Ni (0.0590%); Co (0.0090%); Cu (0.1600%) and the rest iron Fe.

### 2.4. Corrosion tests

The carbon steel samples were submerged in beakers holding 50 mL acid solutions without and with varying concentrations of BBEI and BCEI for six hours at 303 K. The temperature was kept constant throughout by a water thermostat. The samples were submerged, collected, dried, cleaned, and precisely weighed. The inhibitory action of BBEI and BCEI was evaluated using the weight difference, which allowed for the determination of the inhibitory efficiency *η*_W_ (%) by using the following equations:^14^1
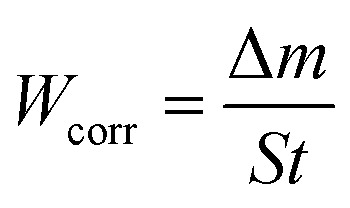
2
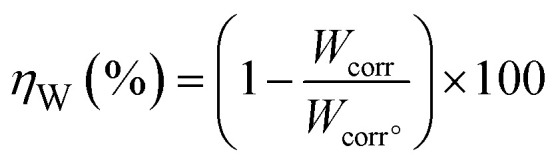
where *W*_corr°_ and *W*_corr_ are the corrosion speeds in the non-existence and existence of an inhibitor, respectively.

Electrochemical measurements and testing are standard approaches for evaluating the corrosion behavior of materials in various situations. In this investigation, the electrochemical measurements were controlled by a potentiostat PGZ100 and corrosion analysis software (VoltaMaster.4). The corrosion cell utilized in the study has three electrodes: a working electrode made of CS with surface of 1 cm^2^, a saturated calomel reference electrode (SCE) and a platinum auxiliary electrode. Before the measurements were collected, and to establish a steady-state open circuit potential (*E*_ocp_), the working electrode made of CS was immersed in the test solution for 30 min at 303 K.

The electrochemical behavior of CS in solutions with and without inhibitor was studied by examining the anodic and cathodic potentiodynamic polarization curves. With a sweep rate of 0.5 mV s^−1^, the electrode potential was automatically moved around the corrosion potential from −800 to −100 mV per SCE. A 1 M HCl solution with varying inhibitor concentrations was used for the measurements. The corrosion current density (*i*_corr_) was calculated by extrapolating the linear Tafel segments from the anodic and cathodic curves. It is important to note that the results obtained in this work on the electrochemical and gravimetric (effect of concentration and temperature) of the uninhibited solution are consistent with those of our previous publication, as all experiments were conducted under identical conditions and using the same equipment.^15^

### 2.5. SEM; AFM; XRD and XPS analysis

The steel surface was analyzed utilizing scanning electron microscopy (SEM), atomic force microscopy (AFM), and X-ray photoelectron spectroscopy after being submerged in a 1 M hydrochloric acid (HCl) solution for 24 hours, except samples that had an optimal concentration of benzothiazolium salts. For our steel, we used the sample preparation procedure outlined in Section 2.2. In order to examine the morphology of the deposited protective layers, AFM images and SEM pictures of the CS surface panels in the existence and nonexistence of two inhibitors BBEI and BCEI were taken.

Utilizing the SPECS-Flex XPS mode and a monochromatic Al-Kα X-ray source (*hv* = 1486.71 eV) with an approximately 3 mm X-ray beam, XPS (X-ray photoelectron spectroscopy) data were collected. A medium surface analysis lens was used to apply a pass energy of 40 eV for analysis, and charge compensation was used to account for charge effects. The binding energy of C 1s (285.0 eV) was the internal reference used. After that, the XPS spectra were deconvoluted using a Shirley baseline and a Gaussian–Lorentzian combination in a nonlinear least-squares technique.

### 2.6. UV-Vis, FTIR and contact angle analyses

The ability to resist corrosion of CS surfaces was also evaluated by ultraviolet-visible (UV-Vis) spectrophotometry before and after the CS sample was submerged for 72 hours to get more insight into the adhesion mechanism of CS surfaces in hostile settings. For this single measurement, analysis was done using a Jenway ultraviolet-visible spectrophotometer.

During the contact angle and FTIR investigations, CS sheets were submerged for a whole day. The Bruker VERTEX 70v model was used to create the attenuating total reflectance Fourier transform infrared spectroscopy (ATR-FTIR). The contact angle measurements were performed using a Dataphysics OCA 50 Micro system, an Up HSC 2000 high-speed camera, and an ES Nano-drop electronic dosing system.

For surface techniques, we used the blank results from previous work published by our team, as we worked under the same conditions.

### 2.7. DFT calculations

For both neutral forms and shapes, the B3LYP/LANL2DZ level was used for DFT computations. For neutral forms in the gas phase, all quantum computations were performed using Gaussian 09 software. In order to have a deeper comprehension of the compounds' activity and corrosion inhibition characteristics, quantum chemical variables such as *E*_HOMO_ (most occupied molecular orbital), *E*_LUMO_ (least occupied molecular orbital) and dipole moment (*µ*) calculated. Hardness (*η*) and softness (*σ*), were also calculated using the following equations:3
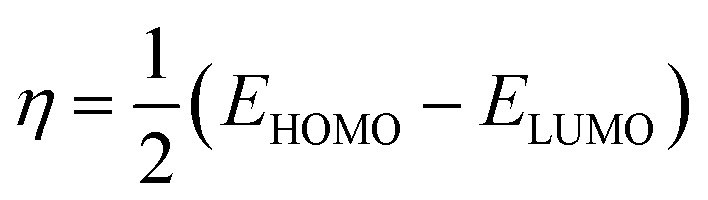
4
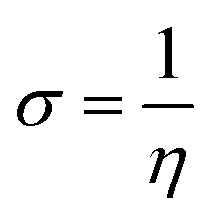


### 2.8. MD simulations

A molecular dynamics (MD) simulation was performed using BIOVIA Material Studio 8.0 software to thoroughly investigate the interaction of the inhibitor compounds BBEI and BCEI with the iron surface. A Fe (110) supercell measuring 12 × 12 and a vacuum slab with a thickness of 5.0 nm were used to simulate the intramolecular interactions inhibitor–Fe-surface in a simulation box measuring 3.0 nm × 3.0 nm × 6.0 nm, without any arbitrary border effects, using periodic boundary conditions to duplicate the Fe-substrate's current effective portion.^16,17^ According to the literature, the Fe (110) crystal surface is the most stable among iron surfaces, including Fe (100) and Fe (111).^18,19^ During the MD calculations, the structure of every system component was optimized using the COMPASS Force Field.^20^ The “Ewald” and “atom-based” summing approaches were used to the “electrostatic” and “van der Waals” non-bonding interactions, respectively.^21^ In the liquid phase, there were 500H_2_O, 5Cl^−^, and 5H_3_O^+^. A 40 Å vacuum layer was put above the liquid layer to prevent surface contact with the repeating periodic planes.^22^ Simulations were conducted at 303 K using an NVT set controlled by an Andersen-thermostat.

## 3. Findings and discussion

### 3.1. Gravimetric study

The inhibitory effect of benzothiazolium salts on carbon steel corrosion in a 1 M HCl milieu was examined using gravimetry without and with various concentrations of inhibitors (BBCEI and BCEI) during a 6 hours immersion period at 303 K. Gravimetric findings for the different inhibitor concentrations studied are given in Table 1. These results include the corrosion rate (*W*_corr_), and inhibitory efficiency (*η*_W_%).

**Table 1:** Weight loss data for CS in 1 M HCl acid medium in the presence and absence of different concentrations of BBEI and BCEI

Inhibitor	*C*_inh_ (M)	*W*_corr_ (mg cm^−2^ h^−1^)	*η* (%)
HCl	1	1.800	—
BBEI	10^−3^	0.035	98.1
	10^−4^	0.061	96.6
	10^−5^	0.092	95.0
	10^−6^	0.210	88.3
BCEI	10^−3^	0.064	96.4
	10^−4^	0.113	93.7
	10^−5^	0.210	88.3
	10^−6^	0.363	79.8

The findings show that, in a 1 M hydrochloric acid solution, every component under investigation prevents steel corrosion. As the inhibitors' concentration rise, the rate of corrosion reduces. The corrosion inhibition, *η*_W_ (%), increases with concentration for the two benzothiazole inhibitors being studied, reaching a highest value (>96%) at 10^−3^ M of inhibitors in both cases. This behavior can be explained by the intra-synergistic effect between the cationic forms of investigated benzothiazolium salts and halide ions (I^−^) on the corrosion-inhibition of CS substrate in 1 M HCl solutions. The large size and the great polarizability of I^−^ facilitate electron pair bonding and therefore enhance the inhibiting power.^23^ Antropov *et al.* explain the increase of the adsorption degree of organic cations in the presence of halide ions by electrostatic interactions between the adsorbed species.^24^ According to IOFA, anions adsorbed on metal surfaces serve as bridges between metal atoms and organic cations, increasing their adsorption.^25^ The anions (I^−^ and/or Cl^−^) most likely adsorb to the metal surface first, generating a negative local charge that attracts both the cationic species of the benzothiazole and the protonated water molecules. This interaction forms a tight triple layer on the metal surface, restricting the solubility of iron ions in solution. It can be concluded that the presence of I^−^ in the solution improves the adsorption of benzothiazolium salts and therefore their inhibition corrosion efficiency in acid solution (Table 1).

On the other hand, BBEI was the most effective inhibitor, with an optimal inhibition efficiency of 98.1% at 10^−3^ M compared to 96.4% for BCEI. This variation in efficacy is caused by the kind of substituent group (chlorine or bromine) on the phenyl ring. The adsorption of the molecule onto the steel surface is facilitated by the para position of bromine, which offers a more even distribution of electrical effects. Chlorine at the *meta* position, on the other hand, lowers adsorption by having a distinct influence on the electron density surrounding the adsorption sites.

### 3.2. OCP study

**Fig. 2:**
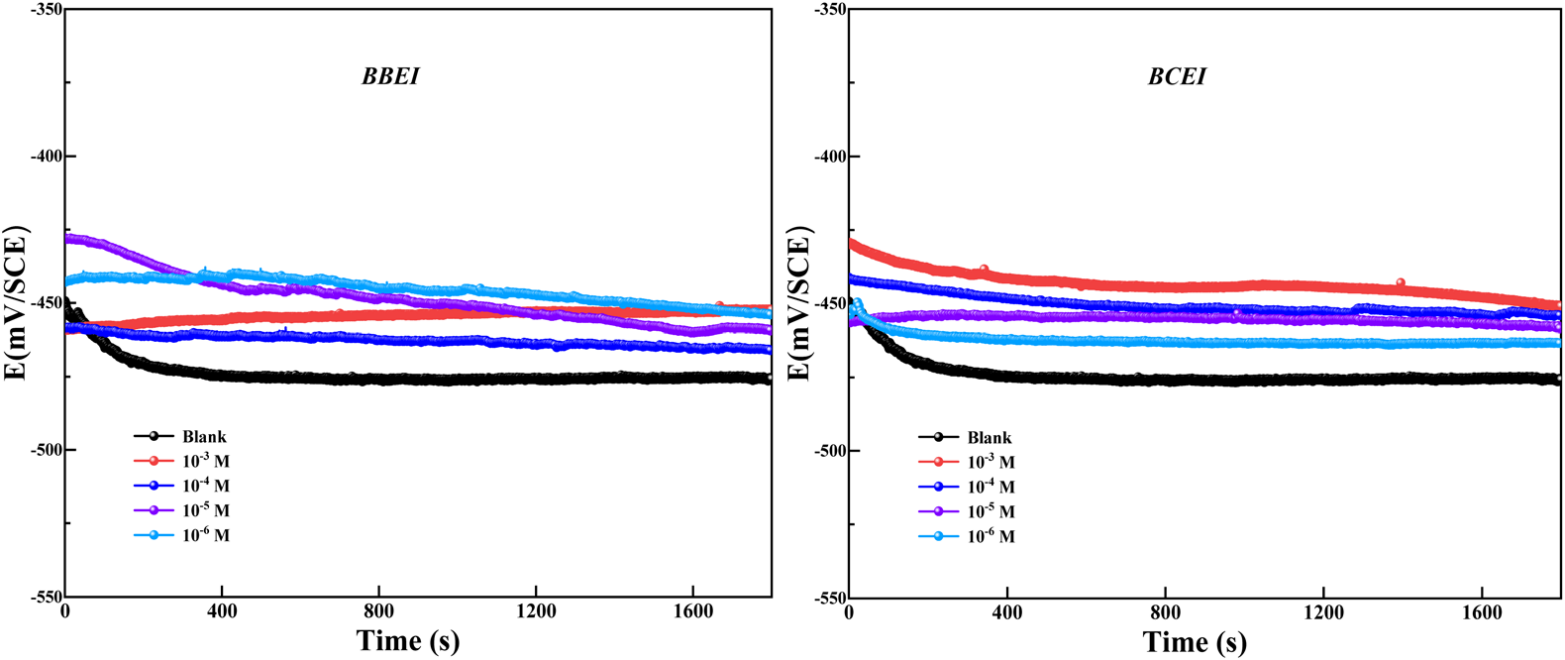
The development of the open circuit potential (OCP) over time for CS in a 1 M HCl solution with and without the addition of BBEI and BCEI inhibitors at 303 K.

In this section of the study, we examine how varied concentrations of the two inhibitors BBEI and BCEI affect the effectiveness of protecting CS against corrosion when exposed to an HCl medium. The open-circuit potential curve of CS in hydrochloric acid with the non-existence and the existence of BBEI and BCEI at 303 K is shown in Fig. 2. The results indicate that the benzothiazole inhibitors (BBEI and BCEI) have a noticeable effect on the cathodic processes, as the OCP values move slightly towards a more positive potential. According to this change, these inhibitors can reduce the corrosion process by slowing down the anodic reactions taking place on the steel surface, particularly the oxidation of CS.^26,27^ After 1800 seconds of immersion in the HCl acid medium, the CS electrode's OCP curves clearly stabilize, indicating that the electrode surface has stabilized.

### 3.3. PDP study

**Fig. 3:**
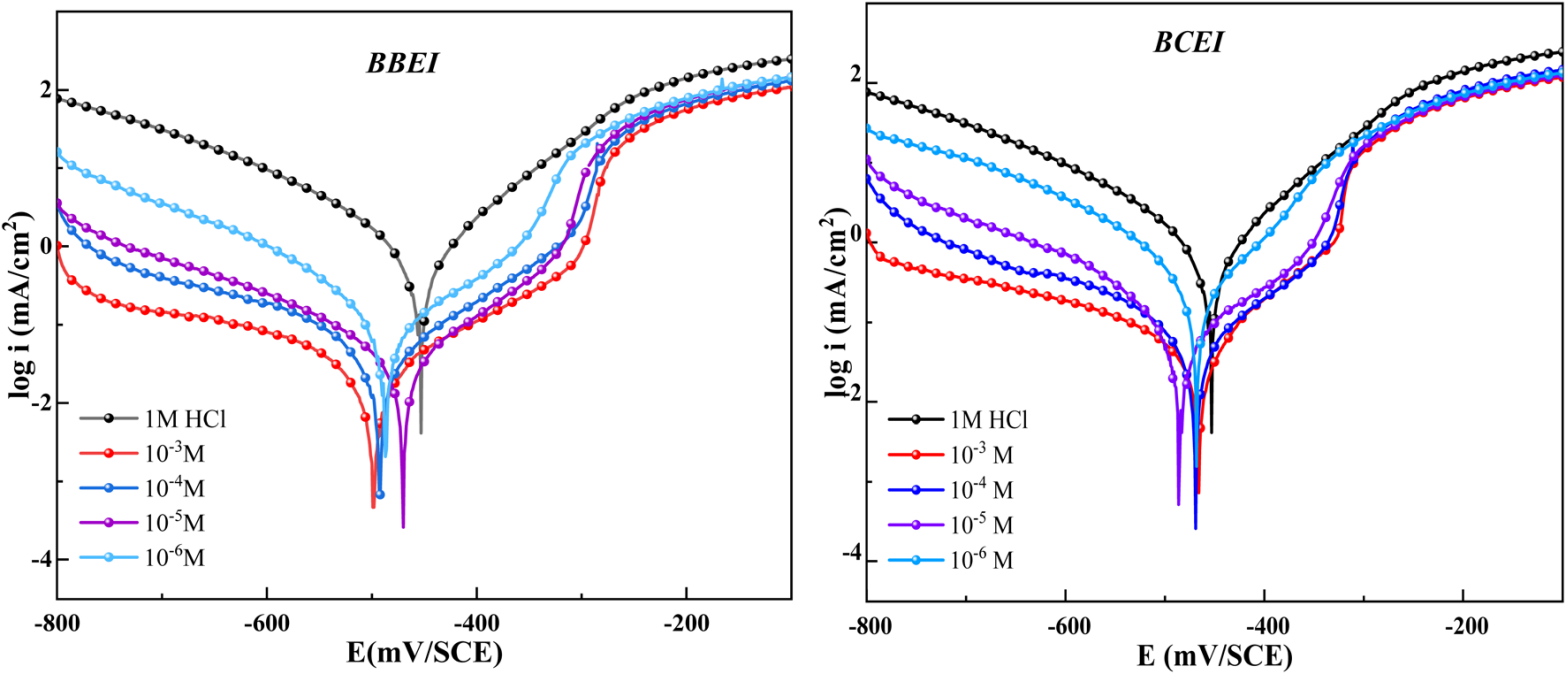
Potentiodynamic polarization curves of CS at 303 K in 1 M HCl with and without BBEI and BCEI.

The ability of BBEI and BCEI inhibitors to prevent corrosion was examined using polarization curves. Fig. 3 shows Tafel plots for CS in 1 M HCl with various BBEI and BCEI doses. Table 2 lists the electrochemical indicators produced from the Tafel extrapolation method, including (*i*_corr_), corrosion potential (*E*_corr_), cathodic (*β*_c_), and anodic (*β*_a_) slopes. The performance ability was calculated using the values of current densities derived from Tafel plots, the latter was calculated using the following formula:5
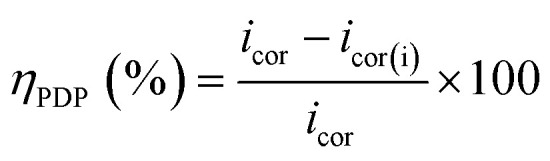
where *i*_cor_ and *i*_cor(i)_ are the corrosion current density values for CS electrodes without and with benzothiazolium salts, respectively.

**Table 2:** Polarization parameters for CS in 1 M HCl solution without and with various concentrations of BBEI and BCEI at 303 K

Inhibitor	*C*_inh_ (M)	−*E*_corr_ (mV *vs.* SCE)	*i*_corr_ (µA cm^−2^)	−*β*_c_ (mV dec^−1^)	*β*_a_ (mV dec^−1^)	*η*_pp_ (%)
Blank	—	456.3	1104	155.4	112.8	—
BBEI	10^−3^	498.5	15.9	136.1	104.3	98.6
	10^−4^	491.0	29.1	115.6	106.0	97.4
	10^−5^	470.1	42.4	165.8	155.9	96.1
	10^−6^	484.6	82.6	95.3	127.0	92.5
BCEI	10^−3^	466.7	41.7	187.6	100.2	96.2
	10^−4^	465.6	56.0	153.5	119.0	94.9
	10^−5^	478.5	70.2	113.8	137.2	93.6
	10^−6^	465.0	185.9	85.0	82.9	83.2

From Fig. 3, it is evident that the existence of both inhibitors significantly reduces the corrosion current density while simultaneously improving the inhibition efficiency. This outcome is caused by benzothiazolium salts molecules clinging to the surface of metal. More inhibitor molecules adsorb and inhibit the active sites on the metal surface as the inhibitor concentration is increased.^28,29^ Additionally, it can be shown that the polarization curves for the BBEI and BCEI compounds have comparable forms, supporting the Tafel law's applicability in the cathodic domain and the discharge of hydrogen protons following pure activation kinetics.^30,31^ The mixed-type character of BBEI and BCEI is confirmed by the less than ±85 mV difference in *E*_corr_ when compared to the sample without inhibitors.^32,33^ Besides, the potential related to the intersection of the two linear portions in the anodic domain, called the potential of desorption (*E*_d_), was found to be a function of inhibitor concentration in both cases of the inhibitor. Indeed, the *E*_d_ values were positively shifted with increasing inhibitor concentration (Fig. 3). The value of *E*_d_ is also related to the amount of adsorbed iodide ions on the iron electrode surface, as explained by Heusler and Cartledge.^34^ The presence of I^−^ ions in corrosive solutions favoured the stability of the organic cation species on the electrode surface, and therefore enhancement in a positive shift of the *E*_d_.

The PDP investigation showed that it is obvious that the *η*_pp_ (%) increased with concentration for both benzothiazolium salts and exhibits excellent inhibitor properties, the best being the BBEI (Table 2). This behavior is consistent with the data obtained by weight loss experiments. Therefore, the ability of the molecule to adsorb on the steel surface was dependent on the kind of substituent group (chlorine or bromine) on the phenyl ring in benzothiazolium salts. This difference in effectiveness could be explained by computational calculations using DFT method approach (see the last part of this work).

### 3.4. EIS study

EIS is a useful tool for evaluating the interfacial characteristics and adsorption behavior of inhibitor compounds. By giving details of the electrochemical process features and kinetics, it provides a thorough understanding of the mechanism of corrosion-inhibition. Additionally, EIS is a non-destructive method that makes it possible to evaluate the behavior of corrosion inhibition in acidic metal solutions. In this investigation, the two synthesized compounds (BBEI and BCEI) had their inhibitory properties assessed using EIS at various inhibitor concentration doses (10^−3^ to 10^−6^ M) at 303 K.

**Fig. 4:**
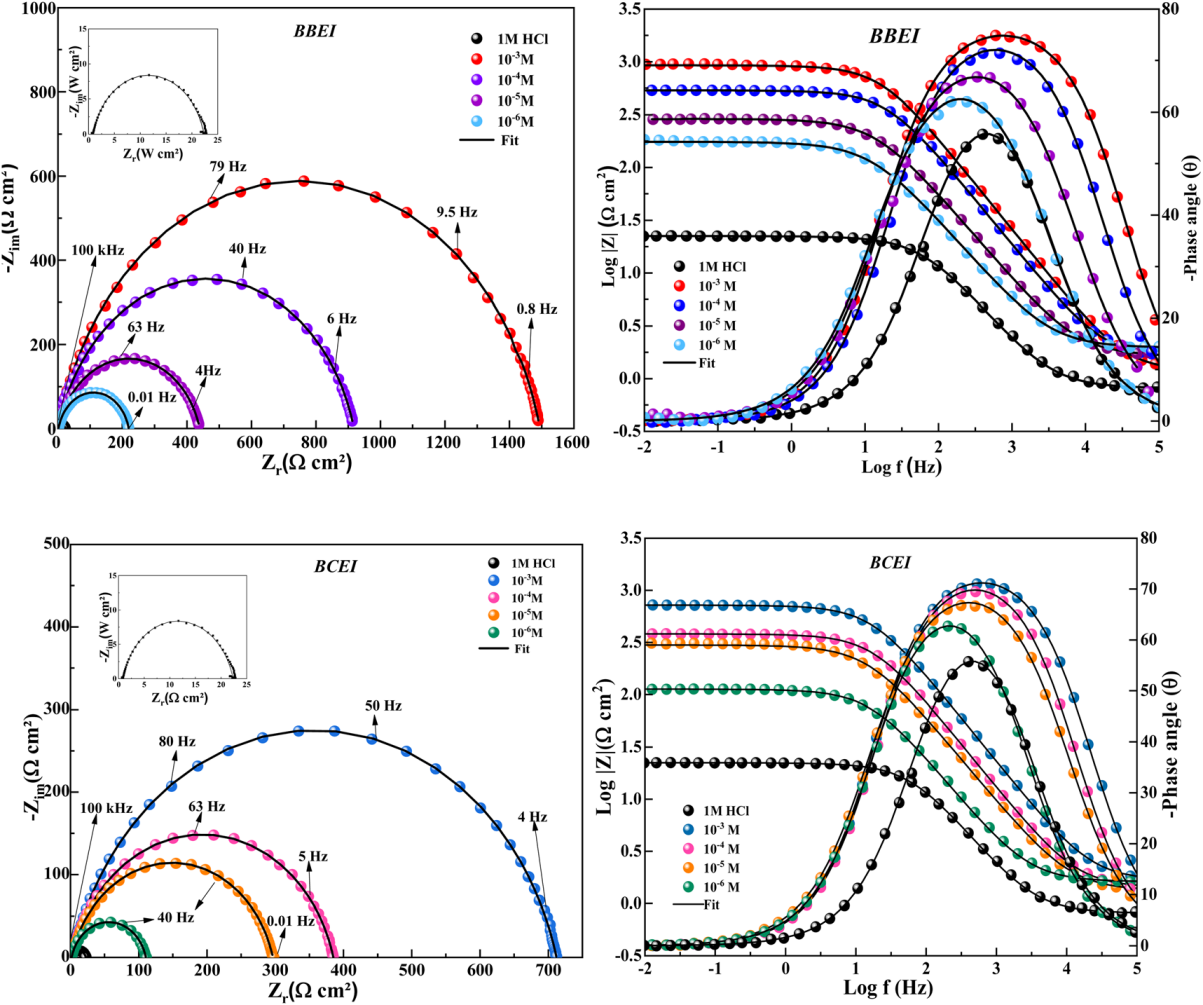
Nyquist and Bode's diagrams of CS in 1 M HCl with and without different BBEI and BCEI concentrations at 303 K.

Fig. 4 shows the presence of a single capacitive loop, indicating that there is a single capacitance loop for BBEI and BCEI because of the inhibition of mass process transfer on the metallic surface caused by the adsorption of inhibitor molecules in acidic solutions.^35,36^ Moreover, the introduction of inhibitor compounds into the corrosive solution had two main effects. However, the size of the capacitive loops increased, reflecting an increase in polarization resistance. This highlights the inhibition of the charge transfer reaction due to the formation of a protective film on the carbon steel surface. Secondly, a shift in impedance towards lower frequencies was observed, indicating an improvement in the interface characteristics between the electrode and the electrolyte. Furthermore, the findings from the Bode representation corroborate the existence of a single time constant, which coincides with the development of the double-layer capacitance. The phase angle values are larger in the presence of the inhibitor than in the blank solution, showing that the CS is better protected from corrosion.^37^

Table 3 demonstrates that as BBEI and BCEI concentrations rise, the double layer capacity (*C*_dl_) declines. However, it was also discovered that when BBEI and BCEI concentration increases, so does the polarization resistance (*R*_p_) value. The process of these compounds adhering to the CS surface can be attributed to a reduction in the dielectric constant and/or thickening of the double layer at the interface between the medium and CS, which validates the adsorption of organic molecules.^38^ Furthermore, the connection between the inhibitors and the CS interface as well as the occupation of free metal sites are indicated by the decrease in constant *A* values upon the addition of inhibitors in comparison to the situation without inhibitors. Additionally, we see from Table 3 that the values of *n* are close to unity, indicating that the interface functions, at least partially, as a capacitive component.^39^ According to the EIS studies, the inhibitor performance sequence is BBEI > BCEI. The increased efficiency can be explained by the bromo substituent in styryl group inhibitor (BBEI) because it facilitates the creation of a denser, more protective passive film than that produced by the chlorinated inhibitor (BCEI). Furthermore, the chi-square *χ*^2^ values are on the order of 10^−3^, displaying a high degree of concordance between simulation and experiment results.^40^

**Table 3:** Impedance parameters for CS in 1 M HCl solution in the non-existence and existence of various doses of BBEI and BCEI at 303 K

Inhibitor	Conc. (M)	*R*_s_ (Ω cm^2^)	*R*_p_ (Ω cm^2^)	10^6^ × *A* (Ω^−1^ s^*n*−1^ cm^−2^)	*n*_dl_	*C*_dl_ (µF cm^−2^)	*χ* ^2^	*η*_EIS_ (%)
Blank	—	0.83	22.0	293.9	0.845	116.2	0.002	—
BBEI	10^−3^	1.1	1494.0	19.7	0.865	11.4	0.008	98.6
	10^−4^	0.9	917.6	23.0	0.857	12.1	0.009	97.6
	10^−5^	1.0	438.8	44.7	0.852	22.5	0.009	95.1
	10^−6^	1.4	218.1	87.6	0.848	43.1	0.006	90.1
BCEI	10^−3^	0.5	709.5	28.2	0.871	15.8	0.007	96.9
	10^−4^	1.6	434.8	31.3	0.870	2.9	0.005	95.0
	10^−5^	1.3	300.2	68.4	0.853	35.0	0.004	92.8
	10^−6^	1.6	108.8	140.0	0.851	67.3	0.009	80.2

### 3.5. Temperature effect

**Fig. 5:**
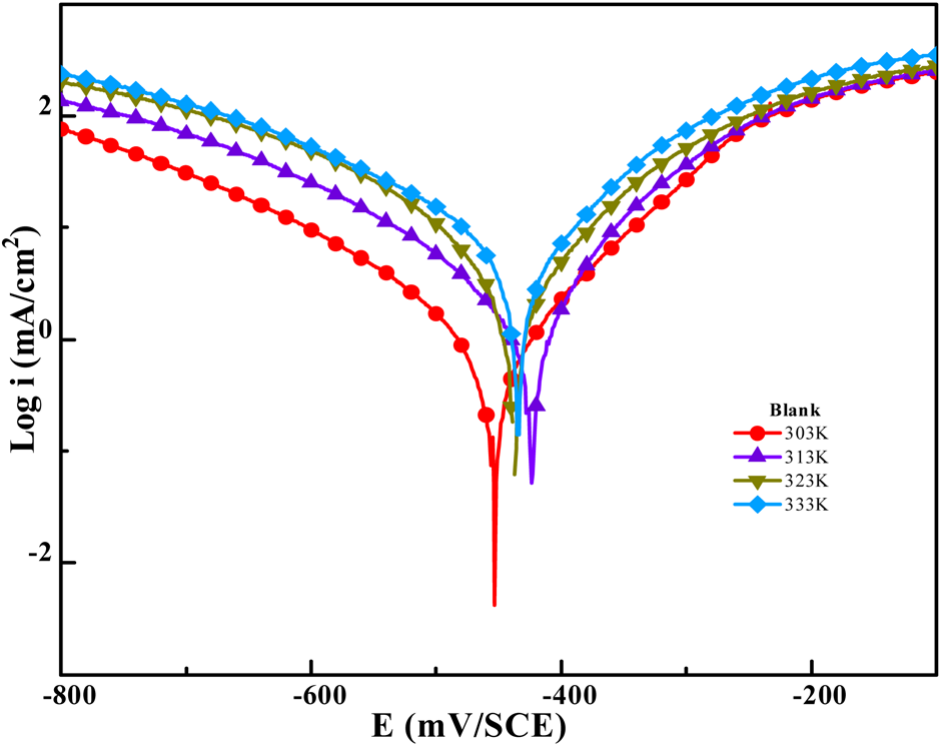
Potentiodynamic polarization graph for CS in 1 M HCl at different temperatures from 303 K to 333 K.

**Fig. 6:**
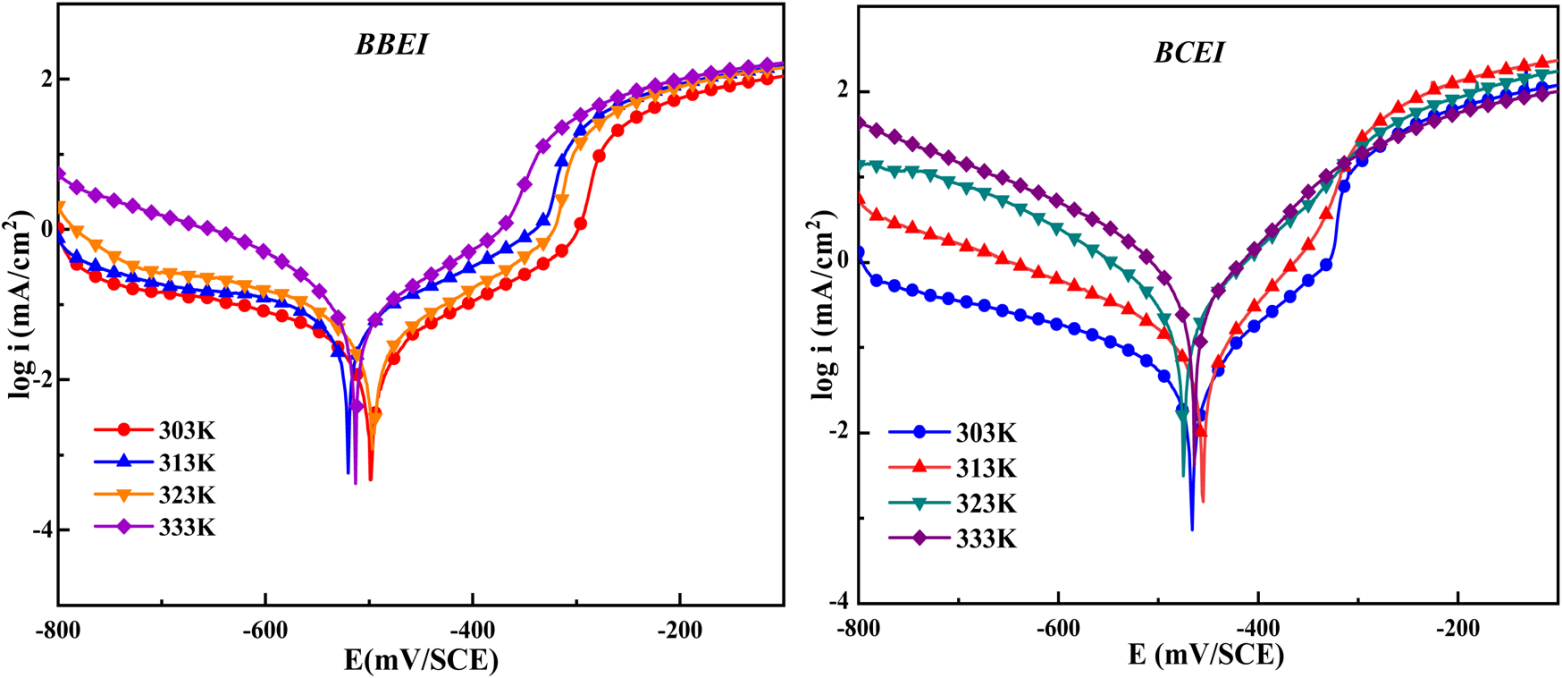
Potentiodynamic polarization graphs for CS with the existence of optimum doses of BBEI and BCEI at different temperatures between 303 and 333 K.

The temperature has a complex effect on the inhibited acid-metal reaction since it can induce the inhibitor to undergo breakdown and/or rearrangement, as well as fast etching and desorption on the metal surface. In 1 M HCl solutions, the temperature-dependent variation in the rate of the corrosion process was examined in the presence and absence of benzothiazolium salts. Investigating the corrosion process activation energy as well as the thermodynamic roles of BBEI and BCEI adsorption. This was achieved by examining the corrosion current's temperature dependency, which was found using the Tafel extrapolation method. Fig. 5 and 6 show the polarization curves for CS electrodes in 1 M HCl in the presence and absence of 0.001 M of BBEI and BCEI in the 303–333 K temperature range.

The current densities in the inhibited medium are smaller than those in the uninhibited medium, which is consistent with our molecule's adsorption behavior, according to an analysis of the temperature data. Table 4 presents the results of raising the temperature from 303 to 333 K. For BBEI, the *i*_corr_ values rise from 15.9 µA cm^−2^ to 162.6 µA cm^−2^, and for BCEI, from 41.7 µA cm^−2^ to 327.8 µA cm^−2^. Moreover, the temperature slowly decreases the inhibitory efficiency. This trend may be clarified through the fact that when temperatures rise, the adsorption/desorption process's equilibrium shifts in favor of the molecules of inhibitors being adsorbed from the CS surface.^41^ As a result, at various temperatures, both compounds continue to exhibit good inhibitory efficacy.

**Table 4:** PDP data for CS in the presence of 0.001 M of BBEI and BCEI inhibitors at various temperatures ranging from 303 K to 333 K

	Temp. (K)	−*E*_corr_ (mV per SCE)	*i*_corr_ (µA cm^−2^)	−*β*_c_ (mV dec^−1^)	*β*_a_ (mV dec^−1^)	*η*_PP_ (%)
1 M HCl	303	456.3	1104.1	155.4	112.8	—
	313	424.0	1477.4	131.3	91.3	—
	323	436.3	2254.0	117.8	91.4	—
	333	433.3	3944.9	134.6	103.9	—
BBEI	303	498.5	15.9	136.1	104.3	98.6
	313	518.9	44.9	179.4	131.9	97.0
	323	495.4	70.4	297.2	85.0	96.9
	333	508.9	162.6	83.1	88.2	95.9
BCEI	303	466.7	41.7	187.6	100.2	96.2
	313	453.6	68.4	126.4	76.8	95.4
	323	471.0	177.0	102.0	75.8	92.1
	333	467.8	327.8	100.3	84.5	91.7

To clarify the impact of temperature on the inhibitory mechanism, a kinetic study was done. Analyzing the correlation between temperature and corrosion current density (*i*_corr_) has made it easier to compute activation slopes between corrosion current and temperature, which in turn has allowed for the assessment of the corrosion process's energy evolution. This was done using the Arrhenius equation both with and without the BBEI and BCEI molecules.^42,43^ The following formula is used to determine the apparent activation energy *E*_a_ based on the *i*_corr_ values:6
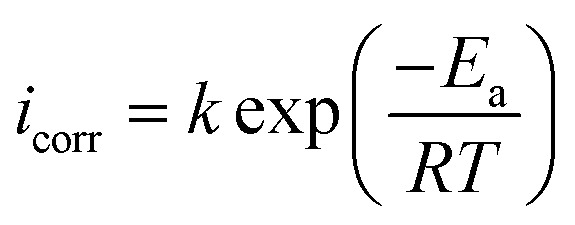
where *T* is the absolute temperature, *R* is the molar gas constant (8.32 J K^−1^ mol^−1^), and *k* is the Arrhenius pre-exponential factor. Additionally, the following transition-state equation may be used to compute the fluctuation in apparent enthalpy (Δ*H*_a_) and apparent entropy (Δ*S*_a_) for the creation of the activation complex in the transition state:7
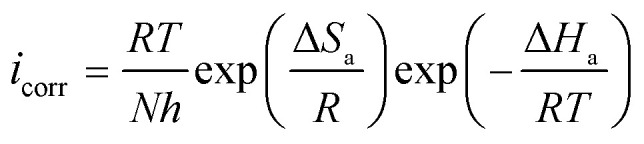


**Fig. 7:**
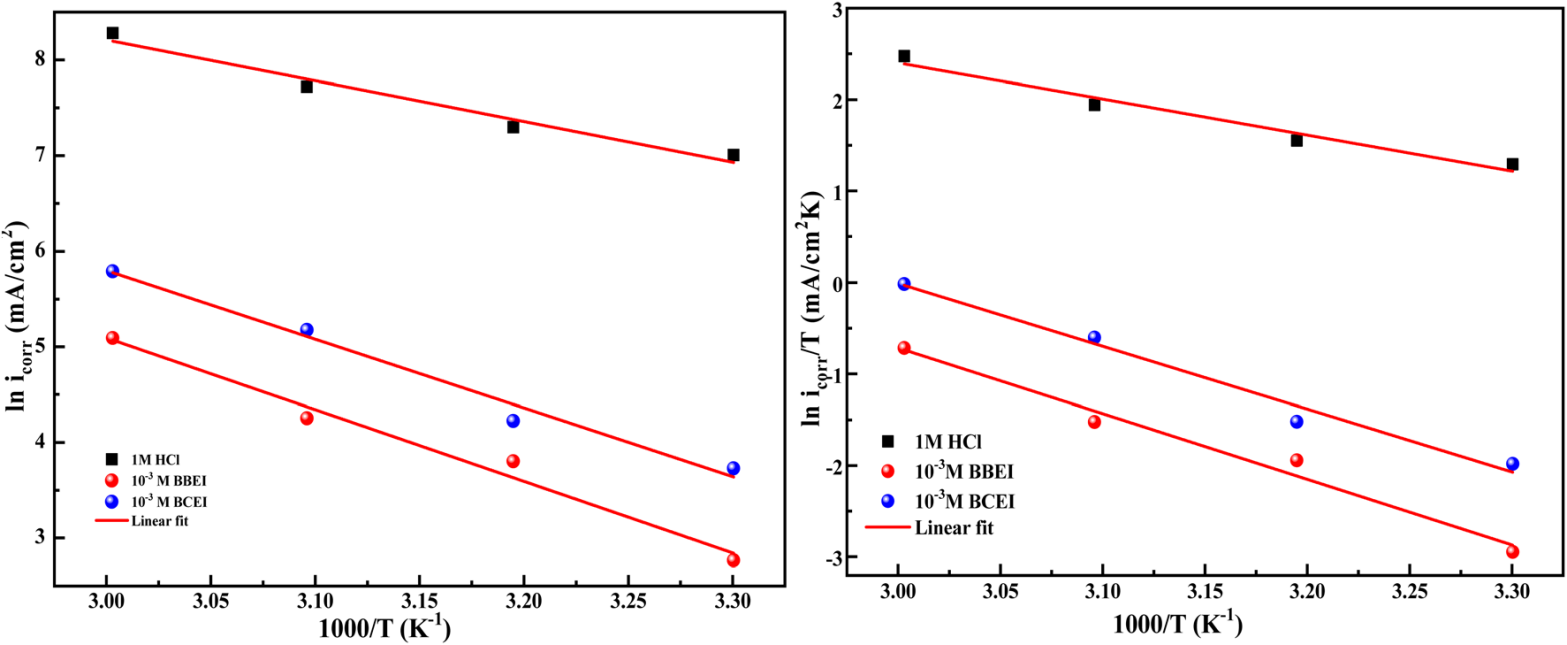
Arrhenius plots for the dissolving of CS in 1 M HCl medium both with and without the ideal BBEI and BCEI inhibitors concentration.

By using linear quadratic fits to relate the natural logarithm of the corrosion index (ln *i*_corr_) to (1000/*T*), activation energies for CS in both inhibited and uninhibited solutions were found. From linear quadratic fits of ln(*i*_corr_/*T*) *vs.* (1000/*T*), the enthalpy and entropy of the activation values were determined (Fig. 7).

The examination of Table 5 reveals that the activation energy values obtained in the uninhibited solution are lower than those obtained in the presence of both BBEI and BCEI. It shows that the drop in metal dissolving in acidic media is caused by a rise in the energy barrier for CS corrosion.^43^ Steel dissolves endothermically, requiring more energy to reach a stable state, as indicated by the positive enthalpy values (Δ*H*_a_). Conversely, the activation entropy (Δ*S*_a_) is positively influenced by BBEI and BCEI presence in the acid solution, although it still stays negative. This implies that the solvent's entropy has increased. This might be the result of both the adsorption of bigger but less disordered BBEI and BCEI molecules and the desorption of water-molecules that had previously been adsorbed on the surface of the metal.^44,45^

**Table 5:** Activation data for CS dissolution in a 1 M HCl, both with and without 0.001 M of BBEI and BCEI inhibitors

	*E*_a_ (kJ mol^−1^)	Δ*H*_a_ (kJ mol^−1^)	Δ*S*_a_ (J mol^−1^ K^−1^)
HCl	35.4	32.8	−79.2
BBEI	62.4	59.8	−24.4
BCEI	59.7	57.1	−26.2

### 3.6. Adsorption isotherm determination

**Fig. 8:**
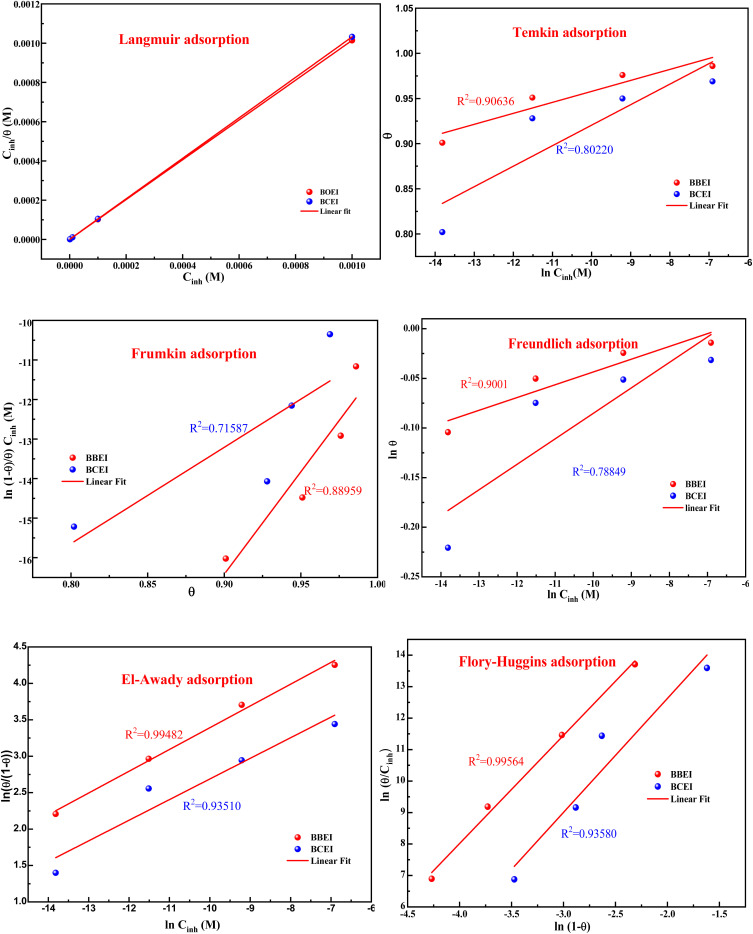
Different adsorption isotherms for CS in 1 M HCl at 303 K for BBEI and BCEI.

To study corrosion inhibition in an acidic medium, one must first understand the adsorption isotherm at constant temperature, which explains the relationship between the concentration of inhibitor in the medium and the level of inhibitor adsorbed on the metal surface. Determining the extent to which an inhibitor adheres to the surface of the metal to create a shielding layer that prevents corrosion is easier when corrosion inhibition is considered, and the adsorption isotherm is known. Different types of isotherms, including Temkin, Freundlich, El-Awady, Flory–Huggins, Frumkin, and Langmuir, can be applied depending on the particular interactions that exist between inhibitors and the surface of metal (Fig. 8).

The correlation coefficient (*R*^2^ ≈ 1), which measures the relationship between *C*_inh_/*θ* and *C*_inh_, in the case of the graph that displays the results, indicates a strong correlation. The Langmuir isotherm model appears to be the most appropriate based on this high association.

The adsorption equilibrium constant (*K*), can be found by graphing the relationship between *C*_inh_/*θ* and *C*_inh_ by utilizing the equation shown below:8
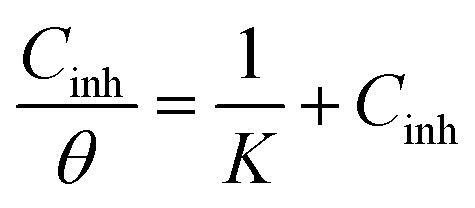


Using the Van't Hoff equation, the Gibb free energy Δ*G*_ads_ can be calculated as follows:9
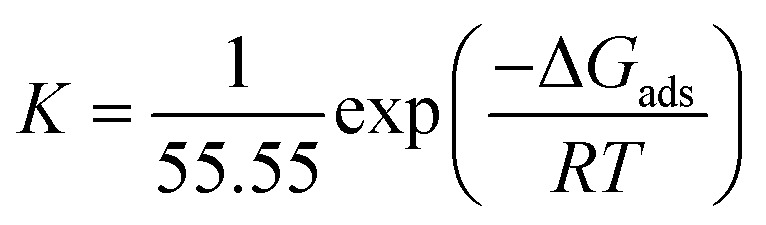


The values of *K* and Δ*G*_ads_ are computed and presented in Table 6. According to *K*, the adsorption coefficient for BBEI was found to be 1 999 192.3 L mol^−1^, whereas for BCEI it was 1 426 963.0 L mol^−1^. The *K* of BBEI are higher than that of BCEI, indicating that BBEI molecules have more noticeable adsorption onto the surface material than do BCEI molecules. Additionally, compared to BCEI, the higher value of *K* for BBEI may suggest that has covered a larger area of the CS sample surface.^46^ The considerable adsorption capability of those inhibitors on the CS surface is demonstrated by the enhanced value of the adsorption equilibrium constant. Additionally, according to several publications, Δ*G*_ads_ values below −20 kJ mol^−1^ represent electrostatic interaction between charged inhibitor molecules and a charged metal (physical adsorption), while values close to −40 kJ mol^−1^ or greater imply charge sharing or transfer from inhibitor-molecules to the surface of the metal to establish a coordinating bond (chemical adsorption).^47,48^ The permanence of the adsorbed layer on the steel surface and the spontaneity of the adsorption process are ensured by negative values of the standard free energy of adsorption, or Δ*G*_ads_. The calculated values for Δ*G*_ads_ are −46.7 for BBEI and −45.8 kJ mol^−1^ for BCEI, suggesting that chemisorption is the typical mechanism of adsorption for both inhibitors BBEI and BCEI on the surface of the metal in 1 M HCl medium.

**Table 6:** Adsorption data of BBEI and BCEI on the CS surface at 303 K

Inh.	Slope	*R* ^2^	*K* (L mol^−1^)	Δ*G*_ads_ (kJ mol^−1^)
BBEI	1.01376	1	1 999 192.3	−46.7
BCEI	1.03121	1	1 426 963.0	−44.6

### 3.7. Spectroscopic studies

**Fig. 9:**
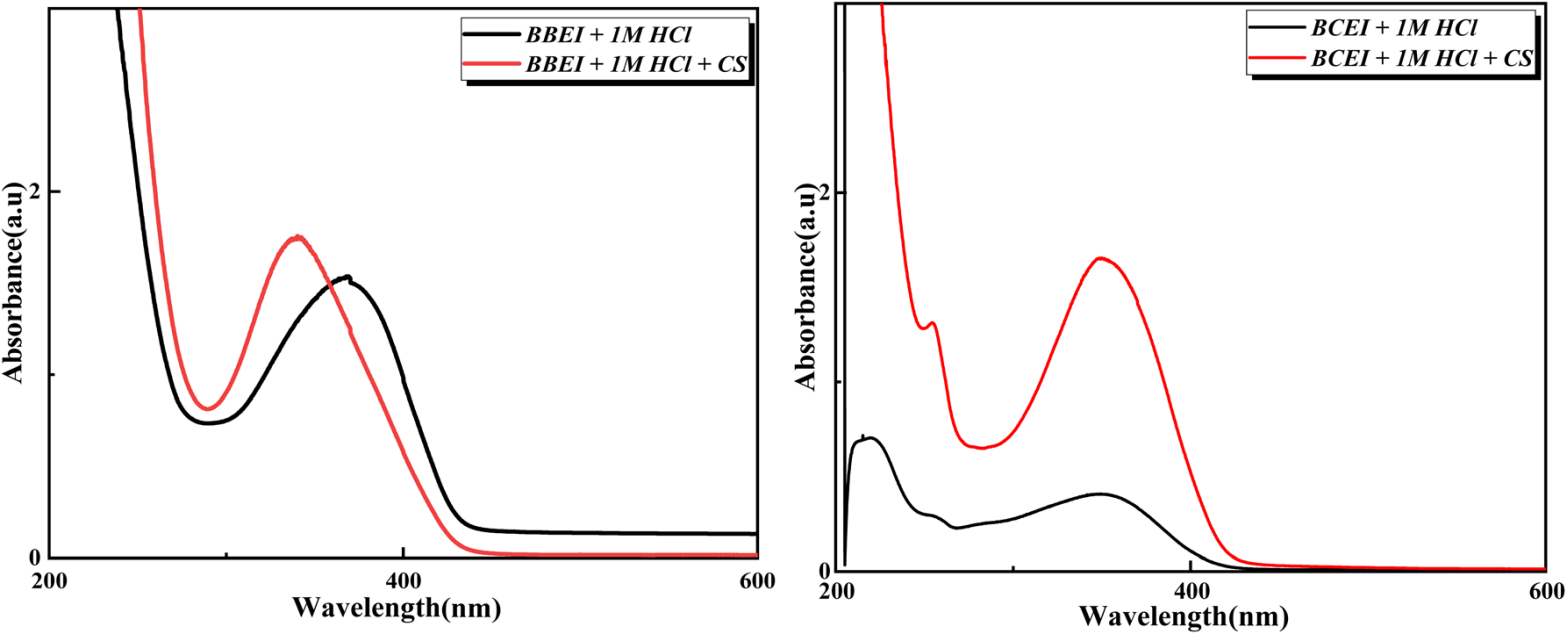
UV-visible absorption spectra of BBEI and BCEI inhibitors before and after immersion in 1 M HCl medium.

Using UV-visible absorption spectroscopy, the adsorption of inhibitor molecules onto the surface of CS was confirmed. To do this, a polished CS coupon was submerged in an inhibitor solution made up of 10^−3^ M of BBEI and BCEI inhibitors for 72 hours. Measurements were made both before and following the produced inhibitor solution's inhibition. The peak shift before and after inhibition, which is related to complex formation on the surface of the metal, is prominently displayed in Fig. 9.

The UV-visible spectrum offers important hints about modifications to the material's electrical characteristics.^49^ In Fig. 9a it is observable that the wavelength shifts from 340.0 to 370.0 nm and the rise in absorbance from 1.53 to 1.74 a.u. point to modifications in the absorbing species surroundings or electronic structure, and in Fig. 9b three bands are seen one at 216.60 nm with an absorbance of 0.590 a.u., one at 256.0 nm with an absorbance of 0.29 a.u., and the final at 353.2 nm with an absorbance of 0.400 a.u. These bands indicate electrical changes that are happening in the steel or between interacting species. However, there are observable changes upon immersion. A band that decreases at 216.6 nm; passivation of the surface of steel may be the source of this band's disappearance. An inhibitory layer may be formed on the material's surface by the employed inhibitors, blocking the absorbing species' ability to interact with the steel.

**Fig. 10:**
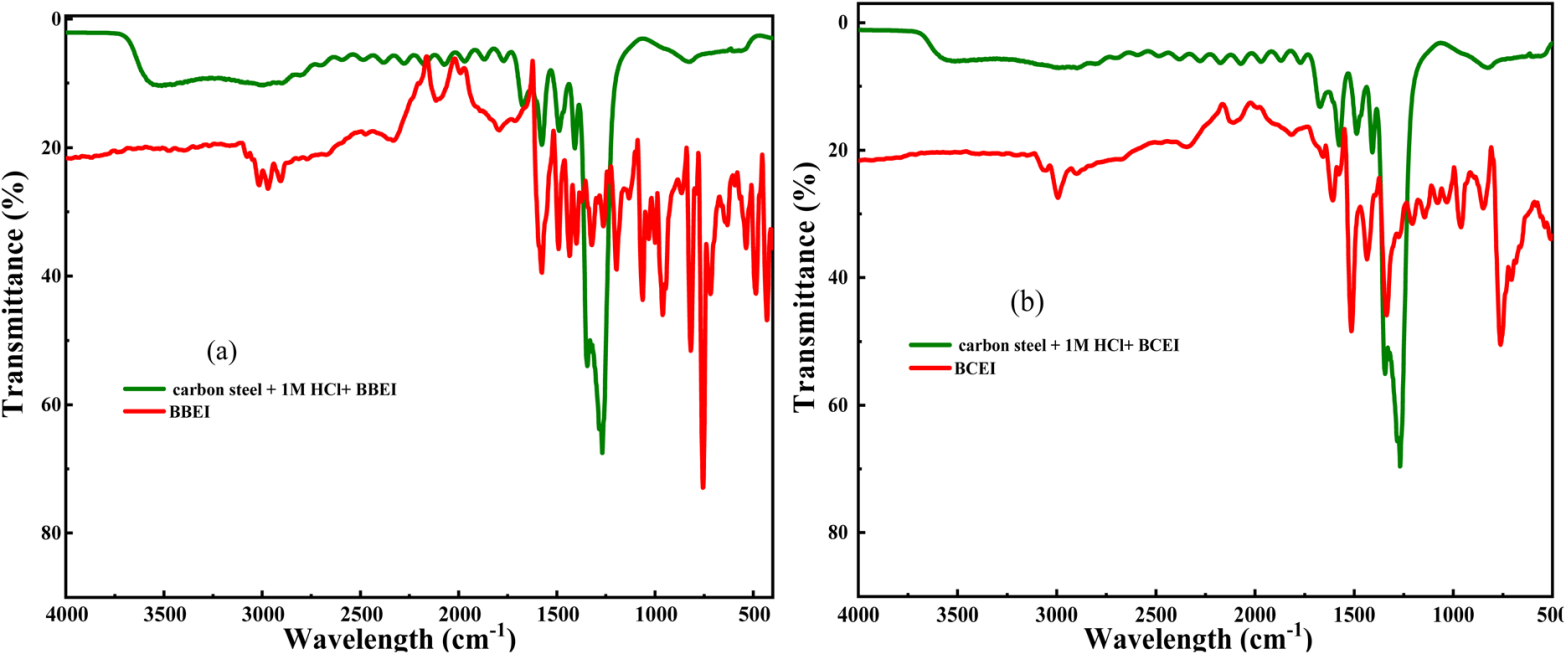
FTIR spectra of the powder inhibitor and the carbon steel after 24 hours of immersion in 1 M HCl medium in the presence of (a) BBEI and (b) BCEI.

Fig. 10 shows the FT-IR spectra of pure benzothiazole and adsorbed benzothiazole films. The pure benzothiazole spectrum shows bands at 3016, 1571, and 743 cm^−1^ corresponding to C–H, C=C, and C–S vibrations, respectively. The benzothiazole-treated samples show bands at 3055, 1554, and 827 cm^−1^ indicating the same functional groups. The samples treated with the inhibitors show prominent bands at 3400 and 1275 cm^−1^ corresponding to the O–H and H–Cl vibrations of the corrosive solution. Furthermore, the appearance of additional inhibitor bands with a small shift or disappearance in the IR spectra of the treated samples suggests a possible association between the inhibitor molecules and the carbon steel.

### 3.8. Surface characterizations

#### 3.8.1. MEB/EDX

**Fig. 11:**
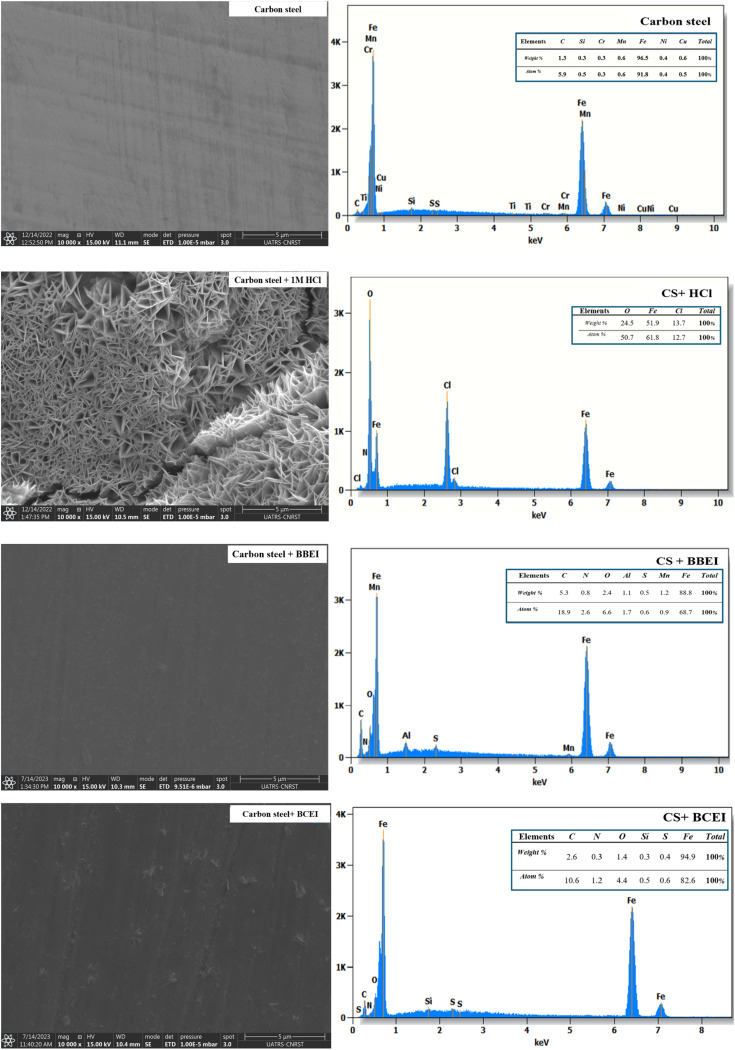
SEM image and EDX spectra of CS before and after one day of immersion in 1 M HCl medium without and with 0.001 M of BBEI and BCEI inhibitor at 303 K.

To validate the acquired outcomes by electrochemical measurements. Qualitative microscopic analyses of MEB have been carried out. Fig. 11 displayed MEB images of the surface of steel in the existence and nonexistence of BBEI and BCEI inhibitors, exposed for 24 hours at 303 K in a 1 M HCl medium. The CS surface is normally clean and unaffected by chemical reactions before it is submerged in 1 M HCl acid. Depending on the metal's reactivity, several occurrences may happen once it is submerged in 1 M HCl acid without the inclusion of two inhibitors (BBEI and BCEI). CS substrate can dissolve and corrode in acid quickly if it is highly reactive, which will produce metal chlorides and release hydrogen gas. Degradation of the metal's surface and material loss may result from this. In order to lessen or stop the acid's corrosive effect on the surface of the metal, two inhibitors have been added to the 1 M HCl acid solution. When BBEI and BCEI were present, the surface of CS showed a noticeable improvement. These inhibitors function as a barrier between the metal and the corrosive environment by covering the metal in a protective coating.^50^ The metal–acid reaction may be impeded by this layer.

#### 3.8.2. AFM analysis

**Fig. 12:**
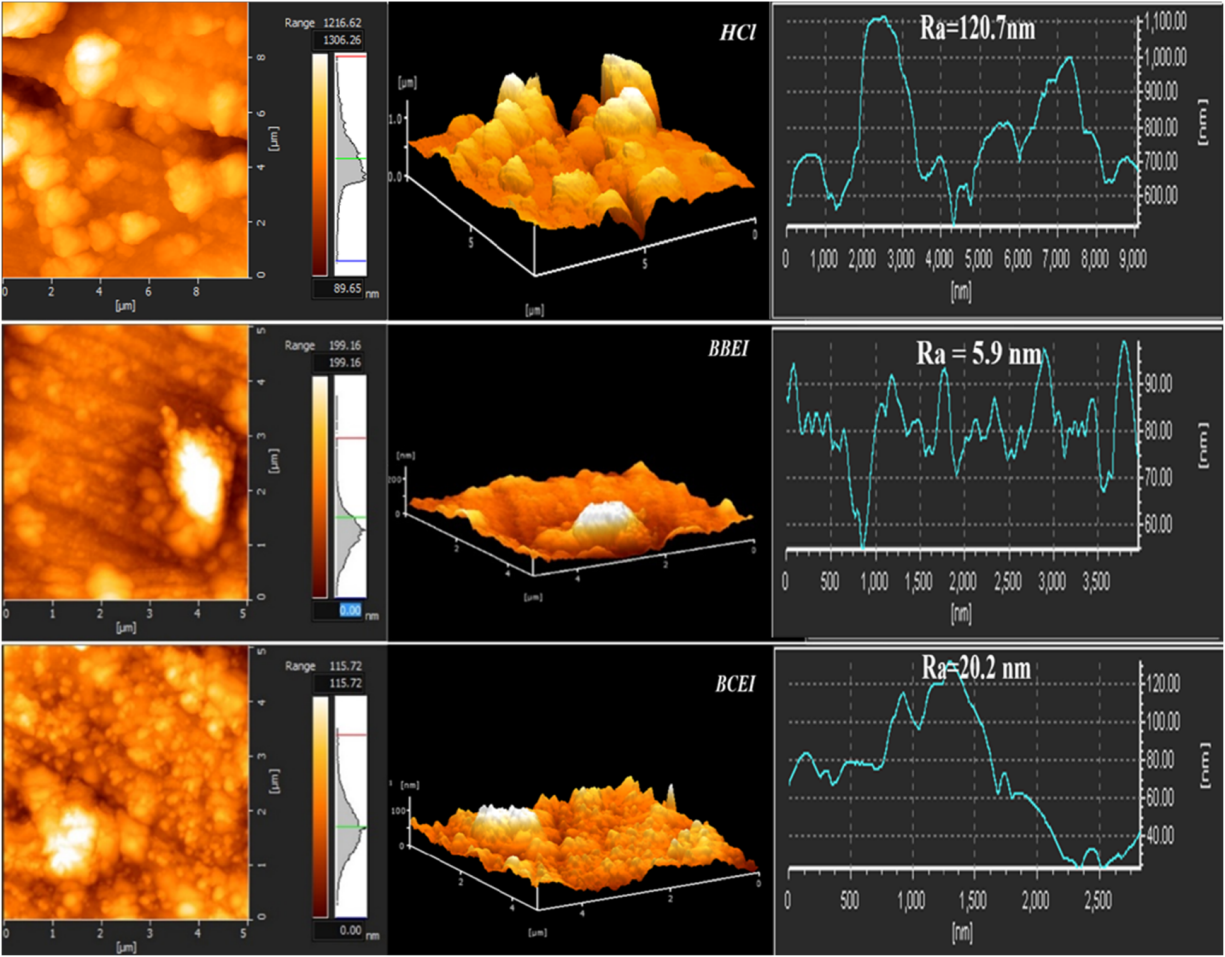
AFM images 2D and 3-dimensional micrographs of CS surfaces (a) in the absence (a) and presence of BBEI (b) and BCEI (c) in 1 M HCl solution.

Atomic force microscope (AFM) was utilized to gather 2D and 3D pictures, allowing for the analysis of the inhibitors' effect on the steel surface as well as the creation of the protective coating (Fig. 12). After a 24 hours immersion of steel surfaces in HCl medium, we examined the surfaces both in the absence of inhibitors and at the optimum concentrations of BBEI and BCEI inhibitors. The steel surface roughens and displays holes and bumps as a result of the acid's attack when it is left in the acid solution alone. Fig. 12 illustrates how surface roughness reduces in the existence of inhibitors. This indicates the inhibitors regulate the corrosion process and that the protective film prevents the steel from corroding any further in the acid solution.^51^ Additionally, when the CS is submerged in a 1 M HCl solution without inhibitors, it has a high average roughness of *R*_a_ = 120.7 nm. In contrast, samples treated with benzothiazolium salts showed significantly less surface damage, with a reduced mean roughness of *R*_a_ = 5.9 nm for BBEI and *R*_a_ = 20.2 nm for BCEI. This considerable reduction in roughness is due to the adsorption of inhibitory molecules, proving their efficiency in shielding carbon steel against corrosion.

#### 3.8.3. Contact angle analysis

**Fig. 13:**
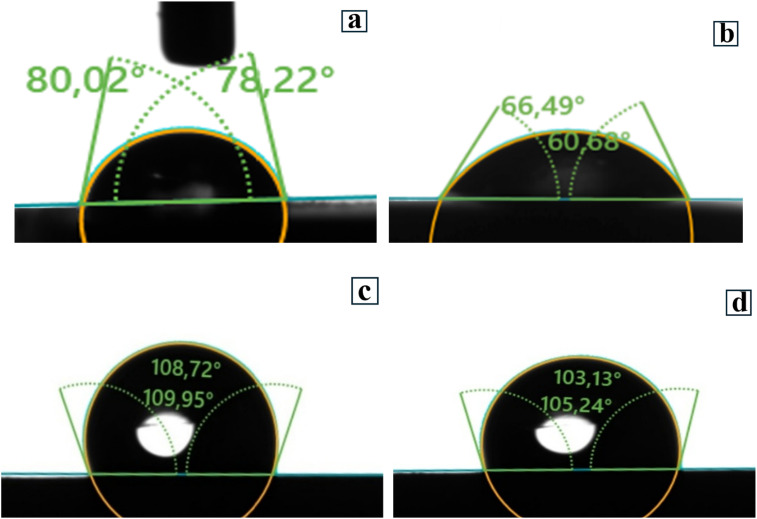
The contact angle tests conducted on CS samples only (a) immersed in 1 M HCl medium in the absence (b) and existence of BBEI (c) and BCEI (d).

Fig. 13 shows the contact angle for the blank samples (a and b) and the samples inhibited by the BBEI (c) and BCEI (d) inhibitors. It was found that the surface of carbon steel was hydrophilic both before and after immersion in 1 M HCl solution, with contact angles of 79.12° and 63.59°. However, after treating the surface with the chemicals BBEI and BCEI, the contact angles increase to 109.34° and 104.19°, indicating a change to a hydrophobic surface. This increased hydrophobicity is related to the formation of a protective layer that resists contact with water. Furthermore, compound BBEI forms a more hydrophobic surface than BCEI, which may be related to the unique structure of the adsorption molecules and the properties of the metal substrate, implying that the adsorption capacity is strongly influenced by these parameters.

#### 3.8.4. X-ray diffraction analysis

**Fig. 14:**
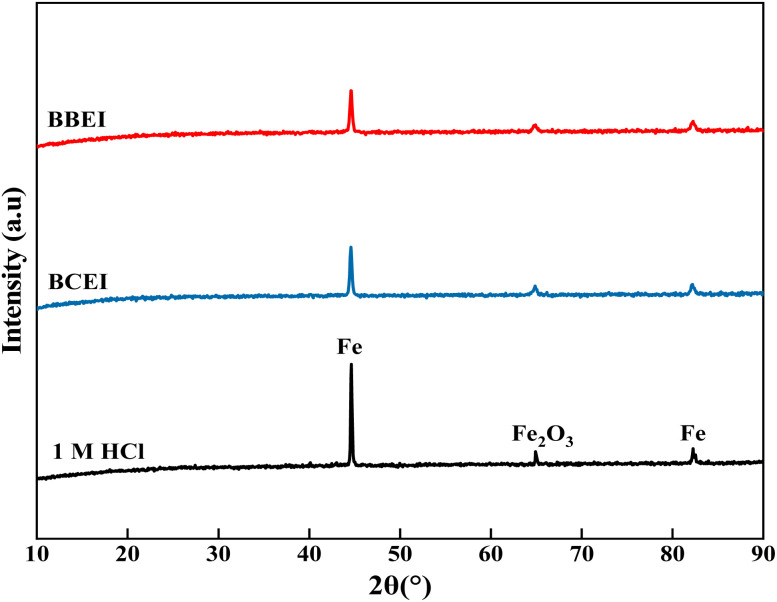
XRD of CS surface in the absence and presence of the inhibitors BBEI and BCEI after 6 h immersion in 1 M HCl.

Fig. 14 shows the XRD diffractograms obtained for carbon steel samples that were submerged in a 1 M HCl solution for 6 hours, both with and without the 10^−3^ M concentration of BBEI and BCEI inhibitors. These diffractograms show considerable changes in the nature and composition of corrosion products depending on whether inhibitors are present or absent. In the absence of inhibitors, the diffractograms show identifiable peaks of metallic iron at diffraction angles 2*θ* of 44.6° and 82.3°, followed by a prominent peak at 64.8°, corresponding to iron oxide (Fe_2_O_3_), which is a common corrosion product in acidic conditions.^52^ These findings suggest active corrosion, with the production of layers of non-protective corrosive compounds. In contrast, in the presence of the BBEI and BCEI inhibitors, the intensity of the iron peaks decreases significantly, indicating that the metal's exposure to the corrosive environment has been reduced. Furthermore, the peaks associated with iron oxide are significantly attenuated or entirely nonexistent, indicating that corrosion products are formed much later than expected. This reduction in corrosive activity is due to the creation of a protective barrier on the steel surface caused by the inhibitors' interaction with the metal.

#### 3.8.5. XPS analysis

**Fig. 15:**
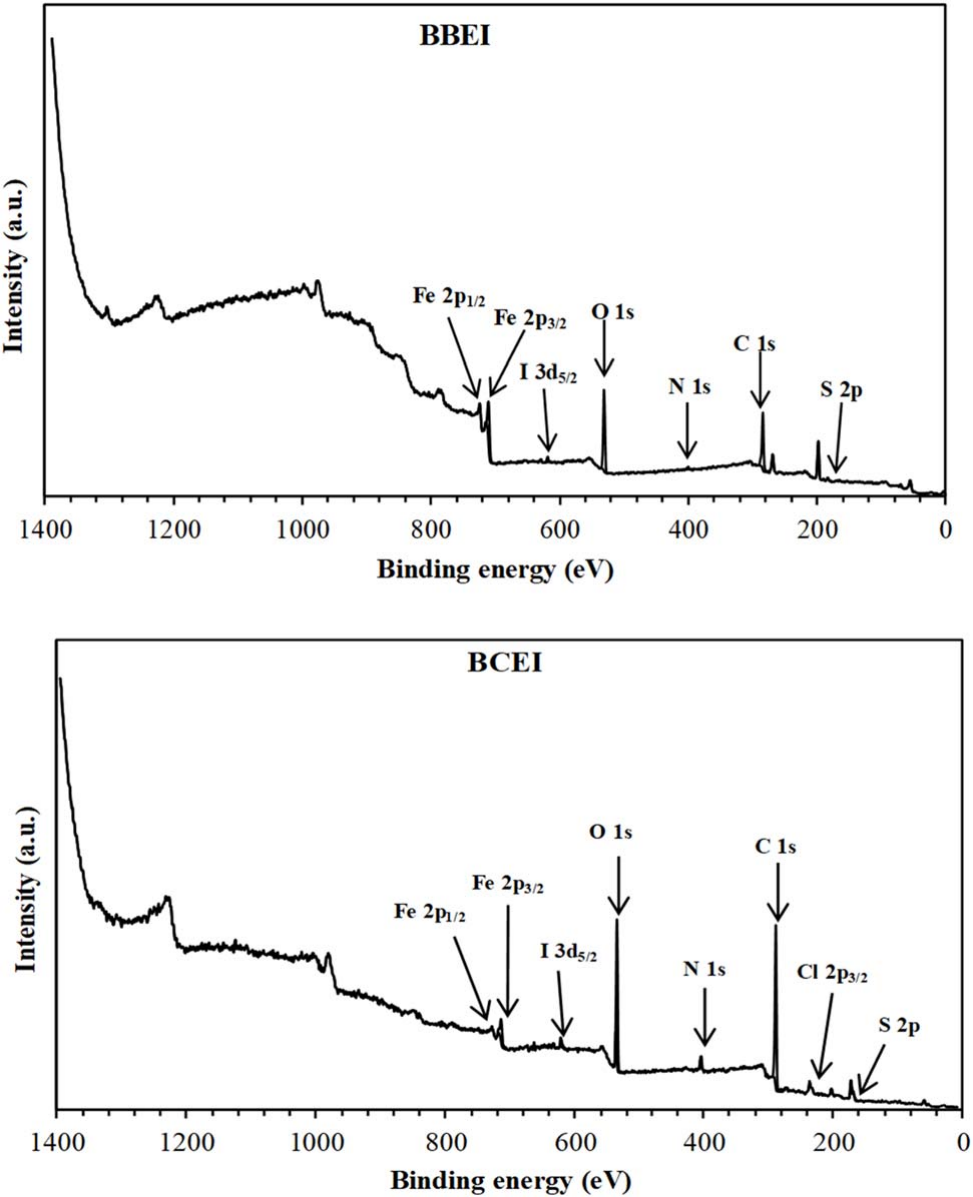
XPS survey spectra of BBEI and BCEI/treated CS after immersion in 1 M HCl.

In order to determine the composition of the organic adsorbed layer on the CS surface in an acid solution after the addition of benzothiazolium salts, the XPS analyses were performed. The resulting XPS wide scan (survey) spectra for CS surfaces after 6 h of immersion in 1 M HCl solution containing 0.001 M of BBEI and of BCEI are shown in Fig. 15. X-ray photoelectron spectroscopy (XPS) revealed the presence of elements N, Br, Cl, and I on the surface of the CS following the addition of the inhibitor in an acid solution, confirming the adsorption of benzothiazole molecules onto the metal, as these elements are not present in the original CS substrate.

**Fig. 16:**
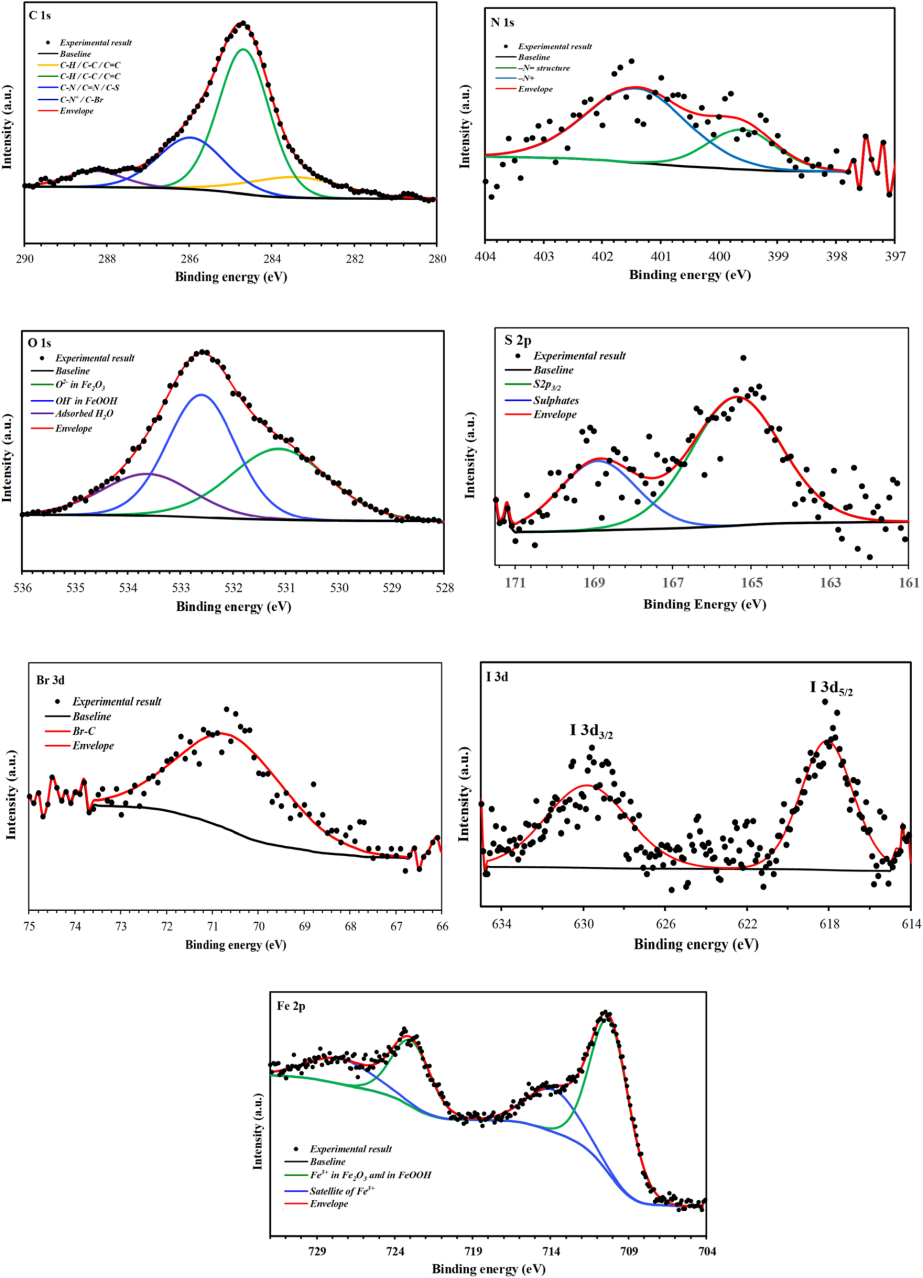
C 1s, N 1s, O 1s, S 2p, Br 3d, I 3d, and Fe 2p high-resolution deconvoluted XPS profiles for CS treated with BBEI in 1 M HCl.

**Fig. 17:**
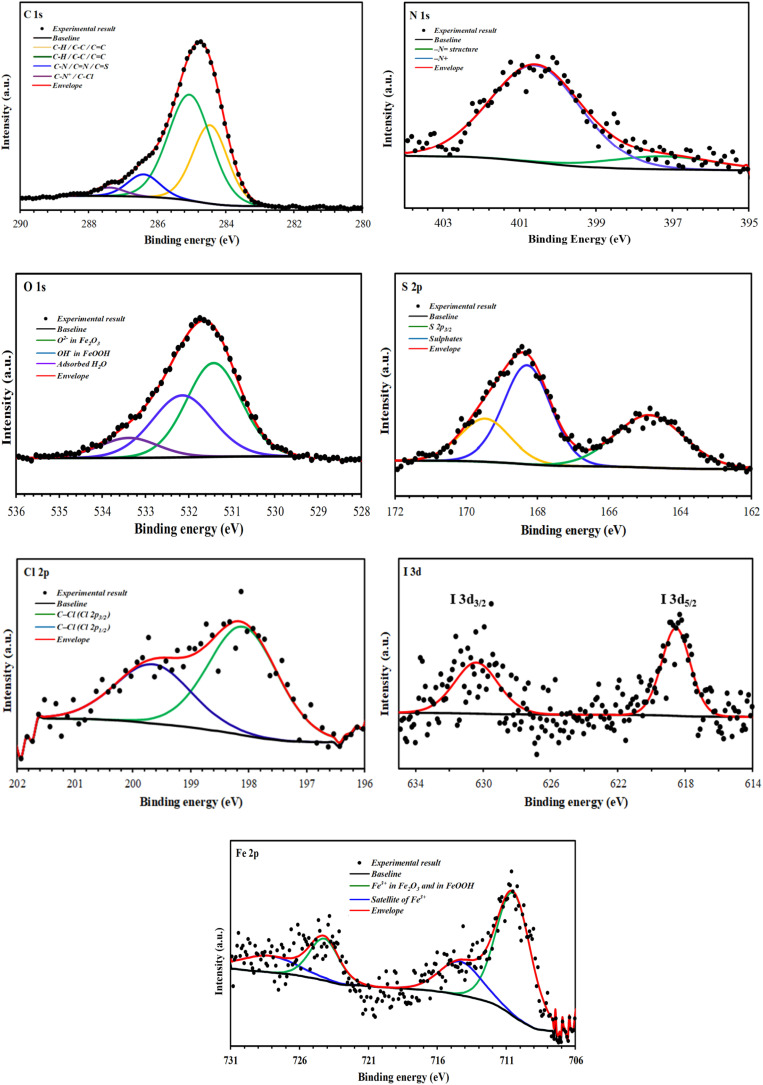
C 1s, N 1s, O 1s, S 2p, Cl 2p, I 3d, and Fe 2p high-resolution deconvoluted XPS profiles for CS treated with BCEI in 1 M HCl.

Fig. 16 and 17 show high-resolution spectra of the core levels C 1s, N 1s, O 1s, S 2p, Cl 2p, Br 3d, I 3d, and Fe 2p obtained using deconvoluted fitting with CASA XPS software. During XPS quantification, a nonlinear subtraction of the Shirley background was performed.^53^ The calculated binding energy (BE, eV) and the associated quantification (%) for each component are summarized in Table 7.

**Table 7:** XPS data for the adsorbed BBEI and BCEI layers formed on the CS surface after immersion in 1 M HCl

Element	Position (eV)	Assignment
**BBEI**		
C 1s	283.4 (14%), 284.7 (54%)	C–H/C–C/C=C
	286.0 (25%)	C–N/C=N/C–S
	288.3 (7%)	C–N^+^/C–Br
N 1s	399.6 (27%)	–N = structure
	401.4 (73%)	–N^+^
O 1s	531.1 (36%)	O^2−^ in Fe_2_O_3_
	532.6 (44%)	OH^−^ in FeOOH
	533.6 (20%)	Adsorbed H_2_O
S 2p	165.4 (68%)	>S structure S 2p_3/2_
	168.9 (32%)	Sulphates
Br 3d	70.6 (100%)	Br–C
I 3d	618.1 (50%)	I–Fe (I 3d_5/2_)
	630.0 (50%)	I–Fe (I 3d_3/2_)
Fe 2p_3/2_	710.2 (77%)	Fe^3+^ in Fe_2_O_3_ and in FeOOH
	713.8 (23%)	Satellite of Fe^3+^

**BCEI**		
C 1s	284.5 (33%), 285.1 (55%)	C–H/C–C/C=C
	286.4 (9%)	C–N/C=N/C=S
	287.4 (3%)	C–N^+^/C–Cl
N 1s	397.2 (14%)	–N = structure
	400.6 (86%)	–N^+^
O 1s	531.4 (51%)	O^2−^ in Fe_2_O_3_
	532.1 (37%)	OH^−^ in FeOOH
	533.4 (12%)	Adsorbed H_2_O
Cl 2p	198.1 (63%)	Cl–C (Cl 2p_3/2_)
	199.6 (37%)	Cl–C (Cl 2p_1/2_)
I 3d	618.5 (53%)	I–Fe (I 3d_5/2_)
	630.4 (47%)	I–Fe (I 3d_3/2_)
Fe 2p_3/2_	710.5 (65%)	Fe^3+^ in Fe_2_O_3_ and in FeOOH
	714.3 (35%)	Satellite of Fe^3+^

After immersion in a 1 M HCl solution containing benzothiazolium salts, the C 1s spectra of the CS surfaces were split into four components of different intensities (Fig. 16, 17 and Table 7). Both inhibitors share the first two components, which are mainly related to the contaminated hydrocarbons and the C–C, C=C and C–H bonds of the carbon structures^54^ (Fig. 1). The C–N, C=N and C–S bonds found in benzothiazolium salts are responsible for the third component, which is located at around 286 eV for BBEI and 286.4 eV for BCEI.^55^ The presence of C–N^+^ in the thiazole fraction, as well as the C–Br bond in BBEI or the C–Cl in BCEI, are largely responsible for the final component, which has a higher binding energy level and lower intensity (288.3 eV for BBEI and 287.4 eV for PPD).^54,56^

The high-resolution for N 1s spectra of protected CS with BBEI and BCEI in 1 M HCl can be fitted into two main components indicating therefore the presence of two chemical states of nitrogen (Fig. 16, 17 and Table 7). The first N 1s component can be attributed to the C–N in the benzothiazole moiety,^57^ while the second one at higher binding energy (401.4 eV in BBEI and 400.6 eV in BCEI) may be associated to the positively charged nitrogen in thiazole moiety.^58^

The deconvolution of the S 2p peaks may be fitted into two components. The first one located at 165.4 eV in BBEI and at 164.8 in BCEI can be assigned to the thiazole ring (–S– structure) as mentioned previously,^59^ while the second one observed around at 168.6–168.9 eV is ascribed to the presence to the sulphate anions (SO_4_^2−^), which can be probably attributed to the degradation of thiazole moiety in corrosive solution.^60^

The Br 3d spectrum of CS substrate covered with BBEI shows one main peak, located at 70.6 eV, and can be attributed to the Br–C bond of 4-bromostyryl group in the BBEI molecule.^58^

Fig. 16 shows a high-resolution spectrum of the Cl 2p core level for the BCEI-coated CS substrate, revealing at least two spin–orbit separation doublets (Cl 2p_1/2_ and Cl 2p_3/2_). The binding energy for the Cl 2p_3/2_ peak is approximately 199.6 eV.^61^ This component is related to the Cl–C bond found in the 2-chlorostyryl group of the BCEI molecule.^61^

I 3d core-level is fitted with at least two spin–orbit–split doublets (I 3d_3/2_ and I 3d_5/2_),^58,61^ with binding energy for I 3d_5/2_ peak lying at about 618.1 eV for BBEI, and 618.5 eV for BCEI (Fig. 16, 17 and Table 7). This former can be attributed to I–Fe bond as mentioned previously.^61^ The detection of I element on CS surfaces corroborates the hypothesis that the anionic species (I^−^) of the ionic liquids improves the adsorption of benzothiazole cationic species and therefore their inhibition efficiencies (synergistic effect).

The Fe 2p spectra of the CS surface coated with benzothiazolium salts reveal a distinctive doublet with peaks at around 711 eV (Fe 2p_3/2_) and 725 eV (Fe 2p_1/2_). A satellite structure on the high-energy side suggests more oxidation of the steel surface. Deconvolution examination of high-resolution Fe 2p_3/2_ XPS spectra shows two prominent peaks (Fig. 16, 17 and Table 7). The first, detected at 710.2 eV for BBEI and 710.5 eV for BCEI, is attributable to the existence of ferric oxides, such as Fe_2_O_3_ (Fe^3+^), and ferric hydroxide species, such as FeOOH,^62,63^ while that located at 713.8 eV for BBEI, and 714.3 eV for BCEI, may be ascribed to the satellite of Fe(iii).^64^ These Fe 2p XPS results are in good agreement with the findings of the O 1s spectra. Indeed, the deconvolution of the high-resolution O 1s XPS spectra for CS surface after immersion in 1 M HCl solution containing benzothiazolium salts consist into three components (Fig. 16, 17 and Table 7). The first one, at 531.1 eV for BBEI and 531.4 eV for BCEI, is ascribed to O^2−^ in the Fe_2_O_3_ oxides.^65^ The second component, observed at 532.6 eV for BBEI and 532.1 eV for BCEI, are attributed to OH^−^, and can be associated to the presence of FeOOH.^66^ The latest at 533.6 eV for BBEI and 533.4 eV for BCEI can be attributed to oxygen of adsorbed water.^67^

### 3.9. Density functional theory (DFT) and results

**Fig. 18:**
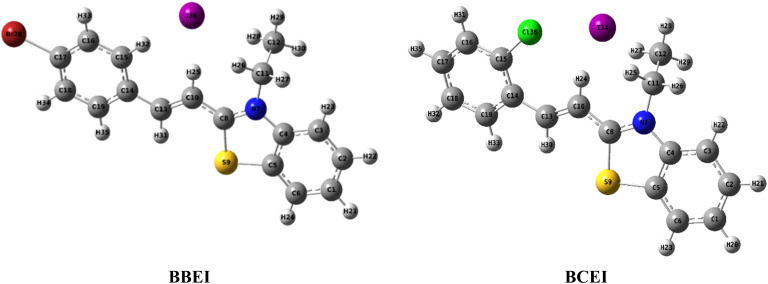
BBEI and BCEI structure optimized.

To study the relationship between the molecular structures of inhibitors and their inhibition properties, theoretical calculations based on DFT were performed, providing valuable insights into inhibition mechanisms and interactions. Fig. 18 illustrates the optimized molecular structures of the inhibitors BBEI and BCEI, determined at the B3LYP/LANL2DZ level.

**Fig. 19:**
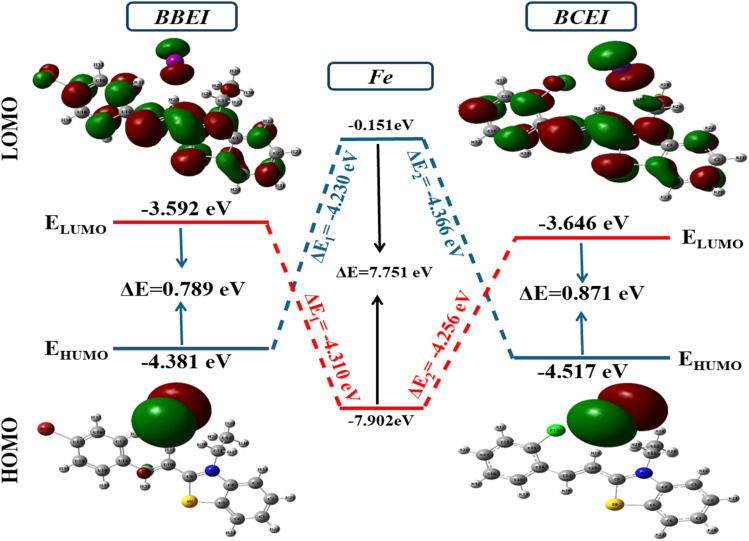
The contour plots of FMO for the BBEI and BCEI at the 3LYP/LANL2DZ.

The distribution of electronic density across these optimized structures is illustrated by the highest occupied molecular orbital (HOMO) and the lowest unoccupied molecular orbital (LUMO) (Fig. 18). The HOMO indicates the ability to donate electrons, while the LUMO highlights the ability to accept electrons. Examination of Fig. 19 reveals that the electronic densities of the LUMO are distributed throughout the molecule, for both BBEI and BCEI, while the HOMO is primarily concentrated around the iodine atoms. This indicates that these regions are actively involved in electron transfer and acceptance during interactions with the studied surface. This observation suggests that the structures of these inhibitors could interact significantly with the surface, highlighting their adsorption potential.

The *E*_HOMO_ and *E*_LUMO_ values provide information about how molecules might interact with a surface, particularly in terms of adsorption. *E*_HOMO_ and *E*_LUMO_ values indicate the energies of the frontier molecular orbitals, with higher *E*_HOMO_ values suggesting that a molecule is more likely to donate electrons to suitable acceptor molecules with an adequate LUMO. Conversely, lower *E*_LUMO_ values imply a higher electron-accepting capacity. According to Fig. 19, for the inhibitors BBEI and BCEI, the *E*_HOMO_ values are −4.381 and −4.517 eV, respectively, and the *E*_LUMO_ values are −3.592 and −3.646 eV, respectively. These values indicate that these inhibitors have a notable capacity both to donate and accept electrons. This suggests that they can interact effectively with the studied metal surface, as their ability to transfer electrons (both donating and accepting) makes them suitable for adsorption on the surface. In other terms, these inhibitors are likely to adsorb well on the surface by forming favorable interactions, which may enhance their effectiveness as corrosion inhibitors.

Based on the HOMO and LUMO energy values for the inhibitors BBEI and BCEI, several quantum parameters have been calculated, including the energy gap (Δ*E*), electronegativity (*χ*), hardness (*η*), chemical potential (*µ*), and softness (*σ*). The results are summarized in Table 8.

**Table 8:** Quantum parameters calculated for the BBEI and BCEI

	Dipole	*E* (eV)	*E*_HOMO_ (eV)	*E*_LUMO_ (eV)	Δ*E* (eV)	*χ*	*η*	*µ*	*σ*
BBEI	14.963	−20350.780	−4.381	−3.592	0.789	3.987	0.395	−3.987	2.532
BCEI	15.209	−20302.834	−4.517	−3.646	0.871	4.082	0.436	−4.082	2.296

The energy gap (Δ*E*) is the principal reactivity parameter; a low value indicates that a molecule can adsorb more easily onto a metal surface. As Δ*E* decreases, the reactivity of the molecule towards the metal surface increases, which enhances the efficiency of this molecule's inhibition. In this study, the energy gap for BBEI and BCEI was 0.789 eV and 0.871 eV respectively. These values suggest that they are highly reactive, which promotes optimal efficiency in their interactions with the carbon steel surface, thereby contributing to optimal inhibition. The tendency of a molecule to donate electrons can be directly assessed by examining its electronegativity (*χ*). A lower *χ* value indicates a greater ease in transferring electrons. For the two studied molecules, the *χ* values are relatively low, at 4.082 eV for BCEI and 3.987 eV for BBEI, suggesting that they are more inclined to transfer electrons to the metal surface. The values of global hardness (*η*) and softness (*σ*) were also calculated. Global hardness (*η*) is related to chemical reactivity: a higher hardness means lower reactivity. The hardness of BBEI and BCEI are 0.396 and 0.436 eV, respectively, which confirms their high reactivity. Conversely, a high global softness (*σ*) is associated with higher reactivity. The softness of BBEI and BCEI are 2.5326 and 2.296 eV, respectively, suggesting that they are effective at adsorbing onto the surface. Moreover, molecules with a high dipole moment tend to adsorb more efficiently onto surfaces due to their enhanced interactions. These interactions promote a more stable attachment of the molecules to the surface and can improve their effectiveness as corrosion inhibitors. Indeed, the dipole moments of BBEI and BCEI are 14.963 and 15.209 D, respectively, which enhances their adsorption onto the metal surface, thus providing protection against corrosion for carbon steel.

**Fig. 20:**
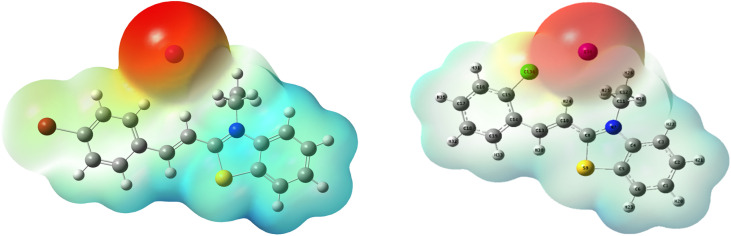
MEP for the BBEI and BCEI molecules.

The MEP identifies the reactive sites of molecules through a color-coded surface representation. Blue surfaces, indicating a positive potential, are associated with low electronic density and are susceptible to nucleophilic attacks. Red surfaces, with a negative potential, are more likely to undergo electrophilic attacks, while green regions correspond to a neutral potential. As illustrated in Fig. 20, the red color is concentrated around the iodine atom in both molecules BBEI and BCEI, indicating that it is particularly favored for electrophilic attacks. In contrast, the hydrogen atoms in the benzyl-rings of the BBEI and BCEI molecules, surrounded by blue color, are more likely to undergo nucleophilic attacks. This suggests that this molecule has a strong tendency to donate and receive electrons when interacting with the surface of carbon steel.

**Fig. 21:**
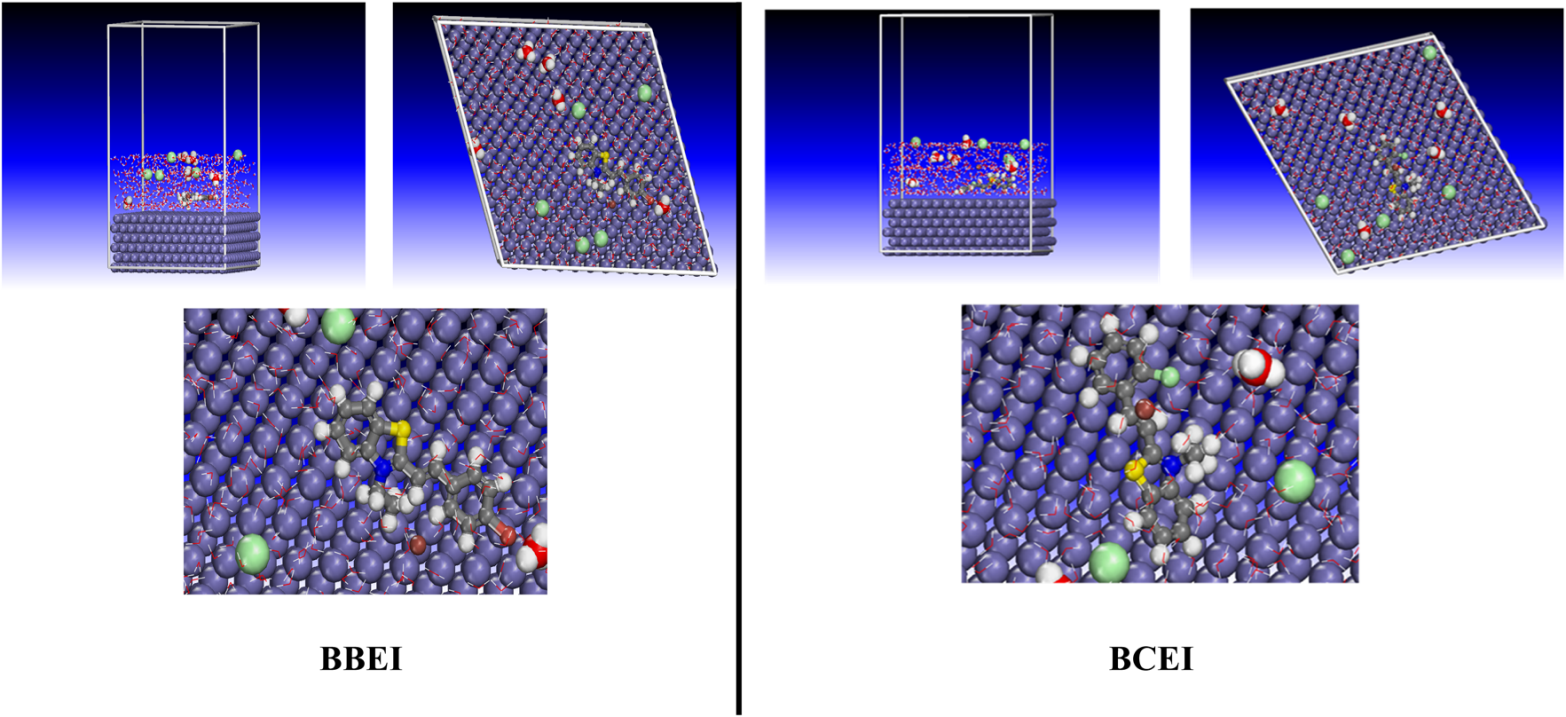
Final snapshots of BBEI and BCEI onto Fe (110) surface acquired from MD simulations.

**Fig. 22:**
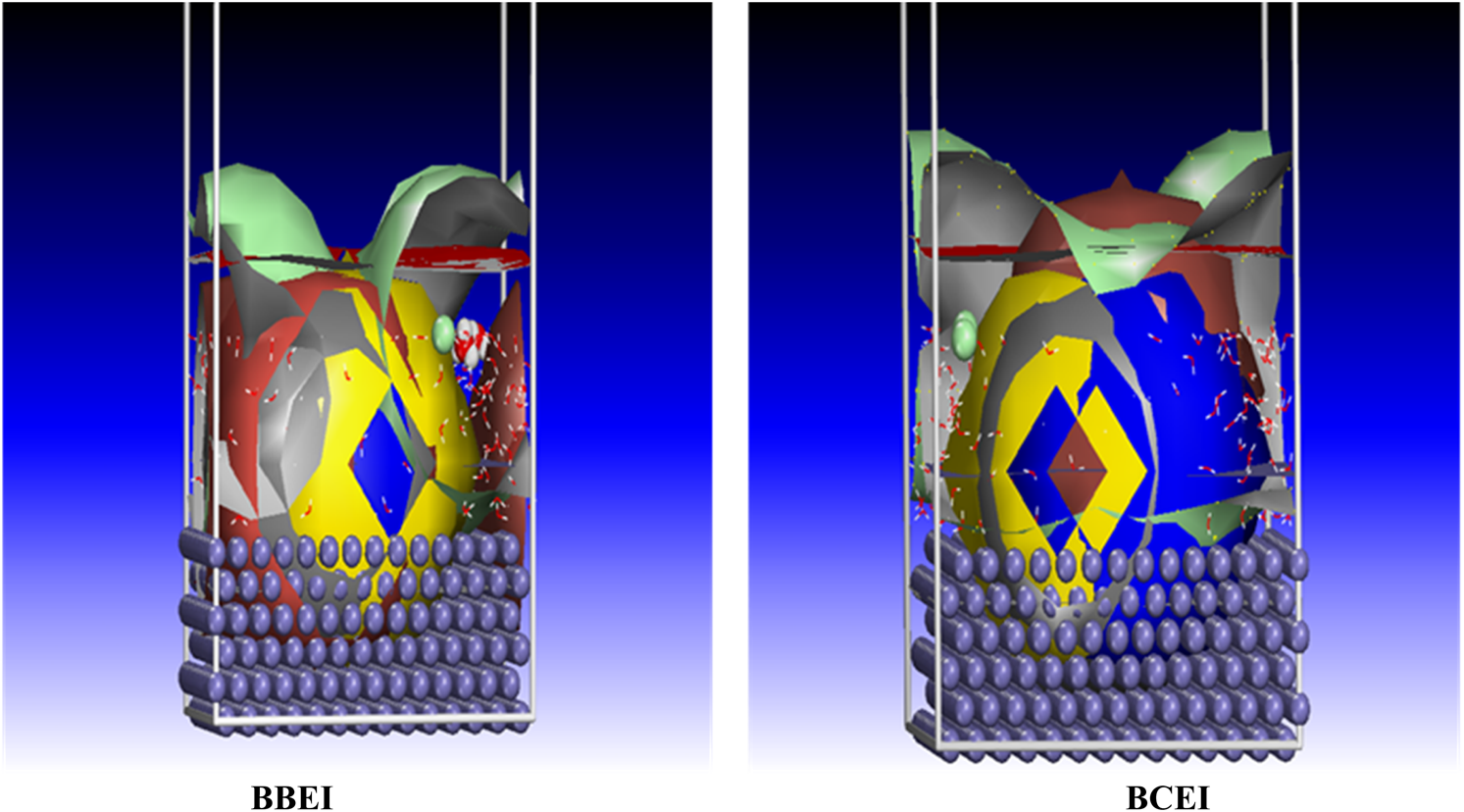
Density field distribution of BBEI and BCEI onto Fe (110) surface acquired from MD simulations.

Quantum chemical simulations of organic compounds' molecular electronic structures are frequently insufficient to completely assess their corrosion inhibitory action.^68^ A more thorough investigation of the inhibitors' direct interaction with the Fe surface is required to better simulate real-world conditions and find the ideal configurations for the inhibitors (*e.g.*, BBEI and BCEI) to adsorb onto the metal surface. In recent years, molecular dynamics (MD) simulation has emerged as a cutting-edge technique for studying metal–inhibitor interactions.^69^Fig. 21 depicts the most stable adsorption configurations of the inhibitors BBEI and BCEI on an iron surface, with the related energies (*E*_binding_ and *E*_interaction_) listed in Table 9. The BBEI and BCEI structures attach closely to the iron surface and align parallel to it (Fig. 21). These adsorption topologies provide better coverage of the iron surface due to the presence of more active sites, such as heteroatoms (S, N) and C=C double bonds, in the BBEI and BCEI inhibitors. Furthermore, the presence of bromine (Br)/iodine(i) in the structure of BBEI and iodine(i)/chlorine (Cl) in the structure of BBEI inhibitor considerably increases their adsorption on the iron surface. These halogen atoms strengthen connections with the metal, increasing the inhibitors' efficiency in preventing corrosion. It is important to note that greater positive values of *E*_binding_ and bigger negative values of *E*_interaction_ shows a strong interaction process between the inhibitor molecule and the metal surface, as well as increased inhibition effectiveness and spontaneity.^70,71^Fig. 22 also shows the adsorption density field distribution of BBEI and BCEI molecules on the Fe (110) surface, which indicates that BBEI and BCEI are effectively adsorbed on the iron substrate through the formation of a strong bond between this molecule and the adsorbent, with preference for BBEI. The MD modeling results show that the interaction energies (*E*_interaction_) for the inhibitors BBEI and BCEI with the iron surface are −1345.8 kcal mol^−1^ and −1241.5 kcal mol^−1^, respectively. Furthermore, the binding energy (*E*_binding_) values were 1345.8 kcal mol^−1^ for BBEI and 1241.50 kcal mol^−1^ for BCEI. The rise in negative interaction and positive binding energies shows that both inhibitors adsorb to the metal surface over time and spontaneously, with BBEI being the most effective inhibitor. These results are consistent with DFT calculations and experimental evidence.

**Table 9:** *E*_interaction_ and *E*_binding_ values obtained from MD simulations of BBEI and BCEI onto the Fe (110) in kcal mol^−1^

System	*E*_interaction_	*E*_binding_
BBEI/Fe (110)	−1345.8	1345.8
BCEI/Fe (110)	−1241.5	1241.5

**Fig. 23:**
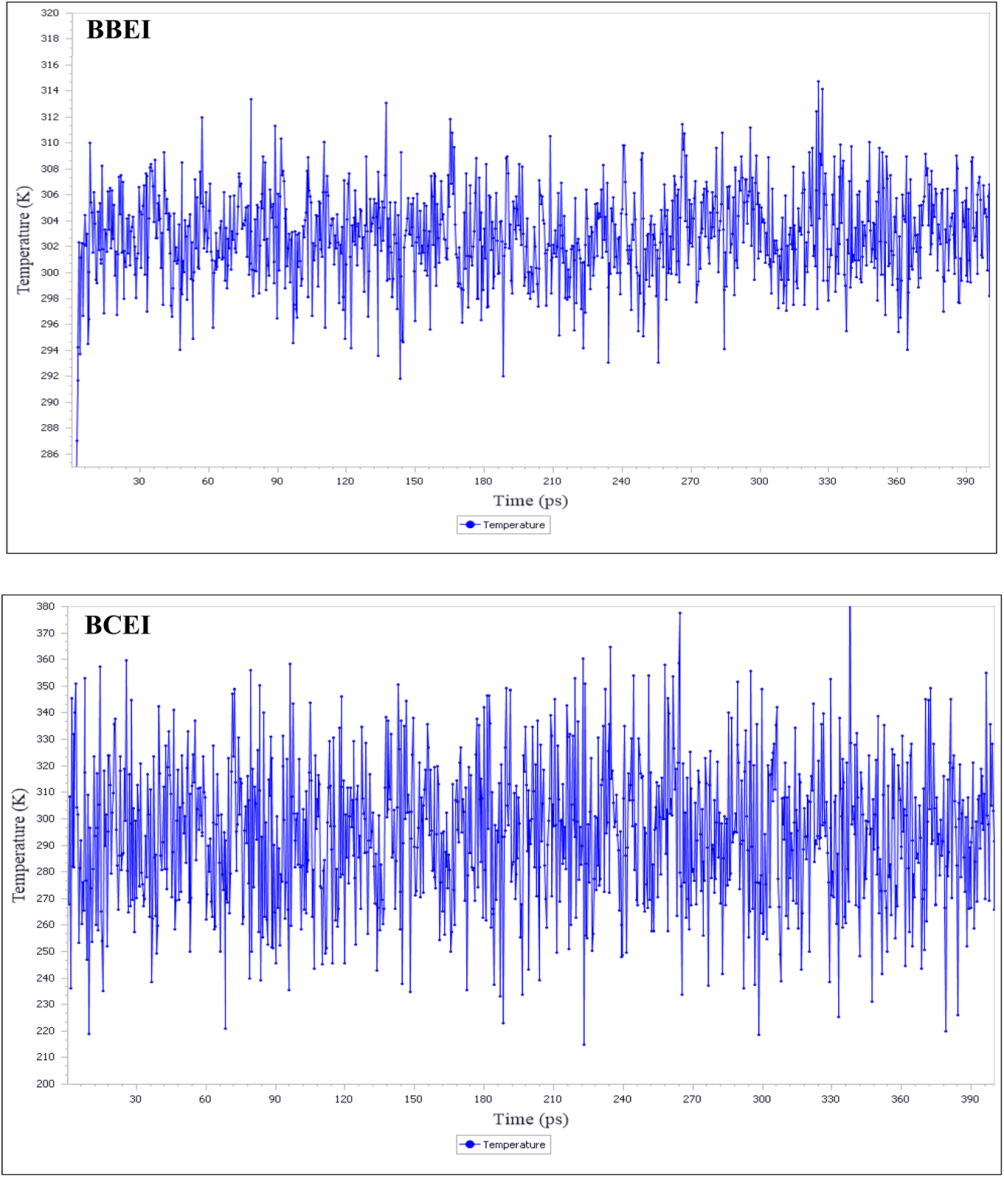
Temperature equilibrium curves for BBEI and BCEI onto Fe (110) surface acquired from MD simulations.

The minor temperature fluctuations seen in Fig. 23 highlight the effectiveness of our system's MD. Given their similar high adsorption negative energy and proximity to the surface, BBEI and BCEI molecules appear to interact significantly with the Fe (110) surface.

**Fig. 24:**
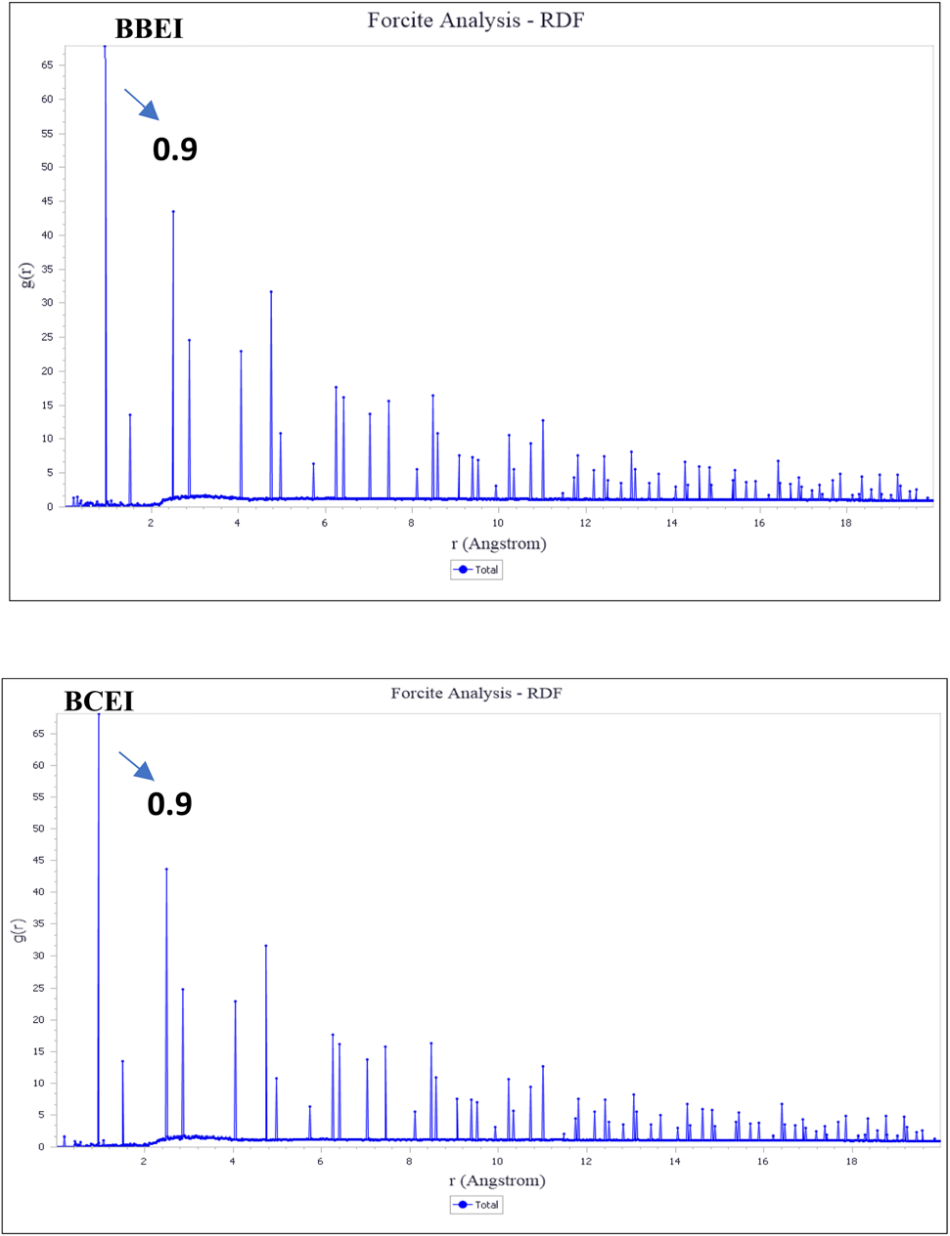
RDFs of BBEI and BCEI over the Fe (110) surface obtained from MD simulations.

The BBEI and BCEI adsorption processes on the Fe (110) surface were examined using RDF to determine if chemisorption or physisorption takes place. The kind of adsorption process is determined by looking for a peak in the RDF graph at specific distances. Distances more than 3.5 Å increase physical adsorption, whereas those between 3.5 and 0.9 Å favor chemisorption.^72^ The RDF results for the BBEI and BCEI structures are shown in Fig. 24. For both inhibitor types, the first significant peak appears at a distance less than 3.5, namely 0.95 Å for BBEI and 0.96 Å for BCEI. Both structures have an initial peak that is smaller than 3.55 A, as seen in Fig. 24, and any subsequent peaks that are larger than 3.5 A are thought to be the result of physical interactions. These are the primary interactions of the simulated title molecules on the first Fe atom layer, proving that BBEI and BCEI do, in fact, prevent the tested metal from disintegrating.

### 3.10. Adsorption mechanism

**Fig. 25:**
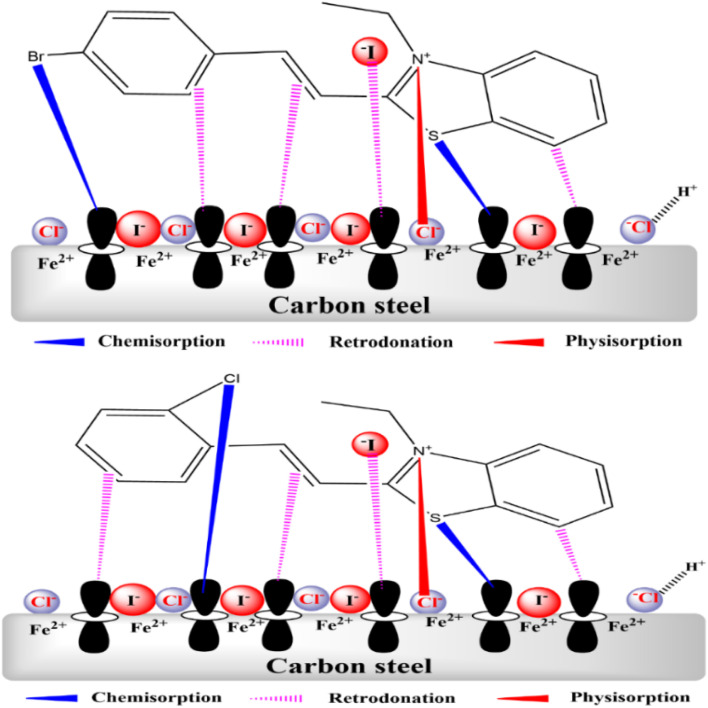
Adsorption mechanism of BBEI and BCEI at the surface of carbon steel.

Inhibitors bind to the metal surface by the process shown in Fig. 25. Due to the absence of electrons, a steel surface absorbs a positive charge when immersed in a hydrochloric acid solution. It then slowly absorbs the corrosive chloride ions, releasing electrons that dissolve the metal at the anode. At the same time, hydrogen ions receive electrons transferred from the steel's hydrated surface that have been adsorbed by chloride ions, resulting in the formation of hydrogen gas at the cathode. The process begins with the accumulation of chloride ions on the surface of the carbon steel, which promotes its dissolution. These ions act as catalysts, causing corrosion of the carbon steel reinforcement by disrupting the bonds between iron atoms on the surface and contributing to the formation of intermediate corrosion compounds. This mechanism gradually causes the iron atoms to detach, thereby accelerating corrosion.^73,74^ However, these electrochemical reactions at the electrodes are significantly affected when BBEI or BCEI inhibitors are added to the solution. Due to their dissociation, these inhibitors are present in the solution in both molecular and ionic forms. On the electrified surface, iodide ions are preferentially adsorbed because they are more voluminous than chloride ions. Through active sites containing S, C=C, and benzene rings, this adsorption promotes the electrostatic attraction of cations to the surface, releasing electrons and forming coordination bonds. The additional electrons from the iron atoms on the surface then occupy the anti-bonding orbitals of the N groups and benzene rings, forming complexes by electrostatic repulsion and retro-donation. This method effectively inhibits corrosion by forming a dense layer of these compounds. In addition, BBE^+^ or BCE^+^ adsorb directly onto the cathodic regions of the substrate, limiting hydrogen ion reduction. Because of their enormous size, these cations displace and replace the water molecules adsorbed on the surface, creating a dense hydrophobic layer that protects the metal from the corrosive environment.

## 4. Conclusions

This study sets out to determine how well two chemicals produced BBEI and BCEI inhibited metal corrosion in an acidic medium. The inhibitors' efficacy against corrosion of CS was examined through both experimental and theoretical analysis.

- It was found that compounds BBEI and BCEI are effective corrosion inhibitors for CS in acidic environments. Polarization curves demonstrate that both inhibitors BBEI and BCEI acted as mixed-type inhibitors. Furthermore, according to electrochemical impedance spectroscopy, the anti-corrosion performance was shown to be in the order BBEI > BCEI, reaching a value of 98.6% and 96.9%, respectively, at 10^−3^ M in 303 K. Therefore, an analysis of how temperature affects inhibition efficiency revealed that it increases as temperature rises. Furthermore, our study has shown that chemisorption is the typical adsorption technique that BBEI and BCEI use on the surface of CS in a 1 M HCl solution. Consequently, tests using UV-visible and FTIR spectroscopy verified the chemical interactions between the inhibitors and the metal surface. In addition, SEM-EDX, AFM, contact angle measurement, and XRD techniques showed the formation of a protective film on the CS. Furthermore, the XPS results support the thermodynamic data by showing that the inhibitors BBEI and BCEI chemisorbed onto the CS surface. The experimental results have been further validated by theoretical investigations conducted on both BBEI and BCEI.

## Conflicts of interest

The authors have declared no conflict of interest.

## Data Availability

No specific additional data is available for this study. All cited data can be found in the manuscript and the supplementary information (SI). Supplementary information is available. See DOI: https://doi.org/10.1039/d5ra07709e.
